# Pyrene-embedded nanohoops: synthesis and dopant engineering for organic solar cells with an enhanced efficiency of 19.96%

**DOI:** 10.1039/d5sc06584d

**Published:** 2026-01-13

**Authors:** Jing He, Wenlong Liu, Siwei Wu, Qi Xie, Zhe Lian, Xiaonan Li, Shengzhu Guo, Ying Wang, Xinjun Xu, Hua Jiang

**Affiliations:** a College of Chemistry, Beijing Normal University Beijing 100875 (P.R. China) ywang1@bnu.edu.cn xuxj@bnu.edu.cn jiangh@bnu.edu.cn

## Abstract

Cycloparaphenylenes (CPPs) have long been a focus of interest for their promising applications in materials science. Herein, we report the synthesis of electron-rich pyrene-embedded nanohoops, [2]OMe-Pyr-[8]CPP and [4]OMe-Pyr-[8]CPP. Single-crystal analysis revealed an oval-shaped cavity with a herringbone molecular packing arrangement, thereby facilitating the formation of extended tubular structures in both derivatives. Photophysical studies revealed that the two pyrene-embedded nanohoops exhibit nearly identical UV-vis absorption and fluorescence emission profiles. We further investigated the potential application of these nanohoops as dopants in organic solar cells (OSCs). When incorporated into D18:L8-BO-based OSCs, [2]OMe-Pyr-[8]CPP enhanced the power conversion efficiency (PCE) from 19.24% to 19.73%. Notably, [4]OMe-Pyr-[8]CPP delivered even better performance, achieving an impressive PCE of 19.96%. These observations indicate that the more electron-rich nanohoop demonstrated superior performance in the present case. These results highlight functionalized CPPs as promising materials for high-performance OSCs, providing an effective strategy for photovoltaic efficiency enhancement.

## Abstract 2

Electron-rich pyrene-embedded nanohoops were synthesized and first investigated as n-type dopants in OSCs. When doped into the D18:L8-BO active layer, they facilitate efficient intermolecular charge transport, yielding a high PCE of 19.96%.

## Graphical abstract

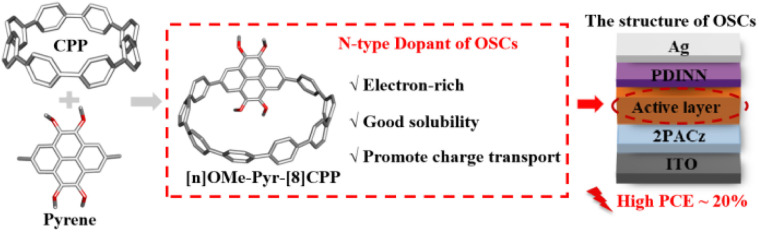

## Introduction

Cycloparaphenylenes (CPPs), a new class of organic conjugated macrocycles, have garnered significant attention due to their radial π-conjugated structures, size-dependent photophysical properties, and good solubility in diverse organic solvents.^1^ The modular synthesis of CPPs provides precise control over their size, shape, and functionalization.^2^ This synthetic versatility further allows for the incorporation of various π-systems into CPP backbones, including donor, acceptor, heteroaromatic, or polycyclic aromatic units.^1*d*,2-3^ Such structural modifications enable fine-tuning of the optoelectronic properties of CPPs, facilitating their tailored application in supramolecular chemistry and materials science.^2–4^ Recent studies have highlighted the potential applications of CPPs in advanced devices including semiconductors,^5^ organic light emitting diodes,^6^ organic photovoltaics,^7^ lithium-ion and organic batteries,^8^ charge transport devices,^9^ and so on.^1,4^ For instance, Bao and co-workers^5^ demonstrated that [6]CPP as an additive significantly enhances both the mechanical and electrical properties of semiconductors. Du *et al.* reported that π-extended poly(cyclo-*para*-phenylene) synthesized from chlorinated CPP building blocks could serve as an effective anode material in lithium-ion batteries.^8*a*^ Moreover, they reported a donor–acceptor (D–A) nanohoop employing the CPP framework as an electron acceptor, which exhibited efficient charge-transfer characteristics.^9*c*^ Our group recently developed naphthalene diimide embedded D–A CPPs showing promise for electron- and hole-transport devices^9*d*^ and an electron-rich tetrathiafulvalene-based nanohoop with unique (*anti*-)Kasha dual emissions and potential in optoelectronic applications.^9*e*^ These findings underscored potential value of CPPs in advanced device technologies.

Organic solar cells (OSCs) represent a promising next-generation energy technology, offering compelling advantages such as low-cost fabrication, lightweight, flexibility, and scalable production.^10^ To realize their full potential, enhancing power conversion efficiency (PCE) remains a critical challenge.^11^ Recent advances highlight molecular doping as an effective strategy to precisely tune the electronic properties of OSCs—whether applied in bulk-heterojunction (BHJ) or layer-by-layer (LBL) architectures, or during device fabrication.^12^ For a component to function effectively as a molecular dopant, several key criteria must be satisfied: (1) incorporation at low concentrations, typically less than 5%; (2) participation in a measurable charge-transfer interaction with the host organic semiconductor; and (3) a primary role in enhancing the concentration of free charge carriers, as supported by the established literature.^12*i*^ This strategy has attracted significant research interest, as it simultaneously addresses multiple performance-limiting factors: enhancing charge transport, improving photogenerated carrier formation, and trap filling.^12,13^ Notably, work by one of our co-authors demonstrated that incorporating poly(9-vinylcarbazole) (PVK) and FeCl_3_ as dopants into LBL-processed OSCs achieved PCE values of 19.05% and 18.12%, respectively.^12*d*,*e*^ Despite such successes, most commercially available dopants suffer from limited chemical diversity and poor solubility, severely restricting their effectiveness.^14^ These limitations underscore the pressing need to develop novel dopants with tailored properties to further advance OSC performance. In this context, CPPs, as a new generation of potential materials for organic electronics,^4–9^ remain largely unexplored as dopants in OSCs, representing a significant challenge that requires immediate attention.

**Fig. 1:**
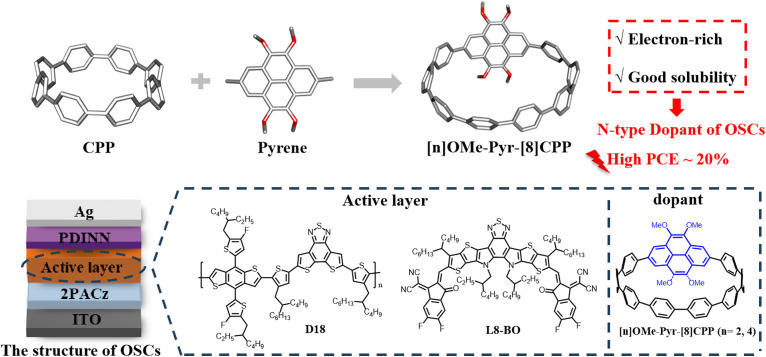
Strategy for the synthesis of electron-rich pyrene-embedded nanohoops and their performance in OSCs.

Building on our ongoing interest in CPP applications in optoelectronic devices and supramolecular chemistry,^2*e*,*f*,3,9*d*,*e*^ we strategically designed electron-rich pyrene-embedded CPP derivatives by integrating pyrene units into [n]CPP frameworks. This molecular engineering approach yielded two novel nanohoops, [2]OMe-Pyr-[8]CPP and [4]OMe-Pyr-[8]CPP, featuring variable methoxy substitution patterns (Fig. 1). The synergistic effects of radial π-conjugated structures of CPPs and electron-rich oxygenated substituted pyrene units may enable their applications as n-type dopant in OSCs. In the present work, we employ molecular doping to precisely engineer the electronic properties of BHJ OSCs, targeting enhanced device performance. To this end, we introduce [n]OMe-Pyr-[8]CPP as an n-type dopant at an optimal concentration into the D18:L8-BO active layer, thereby enhancing charge transport and overall device performance. The incorporation of [2]OMe-Pyr-[8]CPP as a dopant boosted the PCE from 19.24% to 19.73%. Remarkably, devices incorporating the more electron-rich [4]OMe-Pyr-[8]CPP dopant achieved an impressive PCE of 19.96%, representing one of the highest reported values for doped OSCs. Based on the results from device characterization and UV-vis and fluorescence titration experiments, we reasonably conclude that the electron-rich, methoxy-pyrene-embedded CPPs can effectively enhance the intermolecular charge transport between the dopant and the L8-BO acceptor phase in the active layer, thereby contributing to the enhanced charge transport and device performance. These results underscore the potential of CPPs as promising materials for advanced OSC applications.

## Results and discussion

**Scheme 1:**
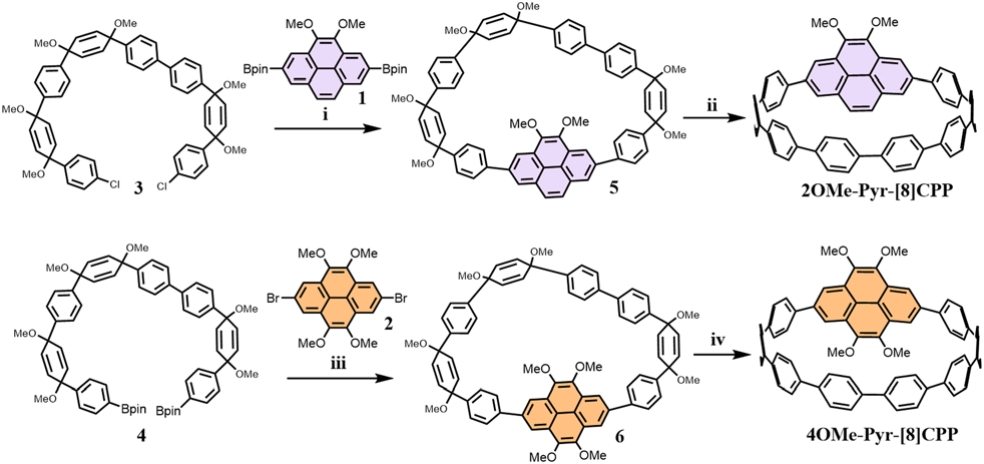
Synthesis of nanohoops [2]OMe-Pyr-[8]CPP and [4]OMe-Pyr-[8]CPP. (i). SPhos Pd G2, K_3_PO_4_, dioxane/H_2_O, 80 °C, 2d, (ii). SnCl_2_, HCl, THF, 30% yield over two steps. (iii). SPhos Pd G3, K_3_PO_4_, dioxane/H_2_O, 80 °C, 2d, (iv). SnCl_2_, HCl, THF, 27% yield over two steps.

We present a synthetic strategy for the construction of crown-shaped nanohoops through Suzuki–Miyaura cross-coupling of sterically hindered components, namely a curved subunit and planar pyrene derivatives, as depicted in Scheme 1. The pyrene-based precursors 1 (ref. 15) and 2 (ref. 16), along with the “C”-shaped building blocks 3 (ref. 17) and 4 (ref. 17) were readily prepared using previously established methods. Initial macrocyclization was achieved through palladium-catalyzed coupling of 2,7-bis(pinacolatoboryl)-4,5-dimethoxypyrene (1) with dichloride 3.This transformation proceeded efficiently using Buchwald's second-generation SPhos (SPhos Pd G2) precatalyst, yielding macrocyclic intermediate 5. Subsequent H_2_SnCl_4_-mediated reductive aromatization yielded [2]OMe-Pyr-[8]CPP in a combined 30% yield over two steps. A similar protocol was employed for the synthesis of [4]OMe-Pyr-[8]CPP, where 2,7-dibromo-4,5,9,10- tetramethoxypyrene (2) was coupled with building block 4. This cross-coupling employed 40 mol% SPhos Pd G3 precatalyst in dioxane/water (5 : 1 v/v) at 80 °C, followed by identical reductive aromatization, affording the target nanohoop in a 27% overall yield.

**Fig. 2:**
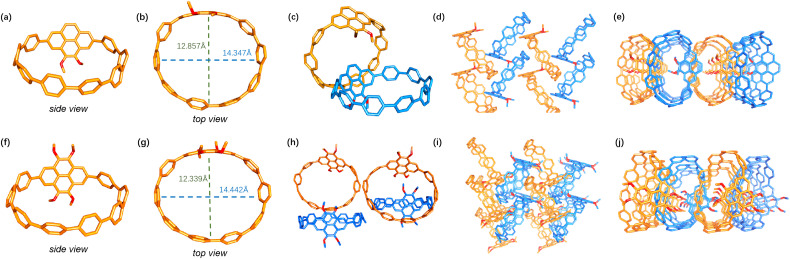
X-ray crystal structures of (a and b) [2]OMe-Pyr-[8]CPP and (f and g) [4]OMe-Pyr-[8]CPP. Crystal packing of (c–e) [2]OMe-Pyr-[8]CPP and (h–j) [4]OMe-Pyr-[8]CPP in the solid state.

The molecular structures of [2]OMe-Pyr-[8]CPP and [4]OMe-Pyr-[8]CPP were unambiguously determined by single crystal X-ray analysis, revealing oval-shaped conformations (Fig. 2).^18^ Both nanohoops exhibit comparable cavity dimensions, with major and minor axes measuring 1.4 nm and 1.2–1.3 nm, respectively. In comparison, [10]CPP adopts a circular shape with a diameter of 1.4 nm.^19^ The elliptical distortion observed in the pyrene-incorporated derivatives is attributed to structural modulation induced by the embedded pyrene units. Crystallographic analysis confirmed that both nanohoops crystallize in monoclinic systems, with [2]OMe-Pyr-[8]CPP adopting a space group of *Pc* and [4]OMe-Pyr-[8]CPP crystallizing in a *Cc* space group. As shown in Fig. 2, the crystal packing mode preserves the characteristics of [n]CPPs,^1*f*^ including both the herringbone alignment and the formation of extended tubular structures.

**Fig. 3:**
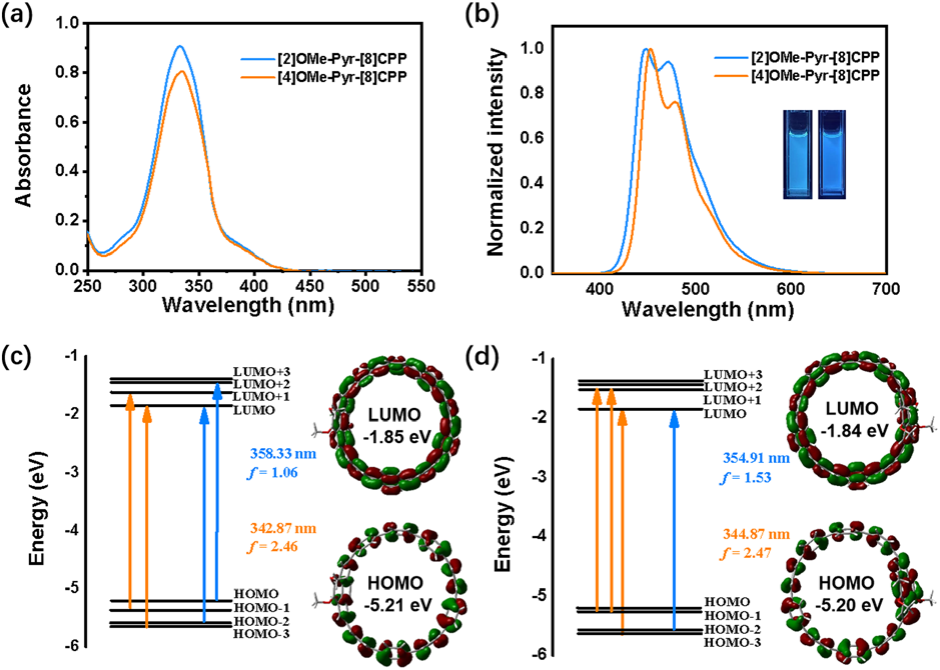
(a) UV-vis and (b) fluorescence spectra of [2]OMe-Pyr-[8]CPP and [4]OMe-Pyr-[8]CPP in dichloromethane (*c* = 1.0 × 10^−5^ M). Photograph of their fluorescence under 365 nm using a UV lamp (inset). Energy diagrams and pictorial representations of the frontier molecular orbitals for (c) [2]OMe-Pyr-[8]CPP and (d) [4]OMe-Pyr-[8]CPP calculated using TD-DFT.

The photophysical properties were studied by UV-vis absorption and fluorescence spectroscopy in dichloromethane at room temperature (Fig. 3). As shown in Fig. 3a, the nanohoops displayed similar absorption profiles within the range of 275 to 425 nm. The major absorption for [2]OMe-Pyr-[8]CPP is located at 333 nm (*ε* = 9.1 × 10^4^ M^−1^ cm^−1^), corresponding to HOMO−1 → LUMO+1 and HOMO−3 → LUMO transitions (Fig. 3c). Introduction of additional methoxy groups results in a slight red shift of the main absorption to 335 nm (*ε* = 8.1 × 10^4^ M^−1^ cm^−1^) for [4]OMe-Pyr-[8]CPP, arising from a combination of HOMO−2 → LUMO+1, HOMO−1 → LUMO+1, HOMO−3 → LUMO transitions (Fig. 3d). Upon excitation at 330 nm, the maximum emission peaks (*λ*_em_) of [2]OMe-Pyr-[8]CPP and [4]OMe-Pyr-[8]CPP were observed at 447 nm and 453 nm, respectively (Fig. 3b). The absolute fluorescence quantum yield was determined to be *Ф*_F_ = 23% and 19%, respectively. These values are lower than that of [10]CPP.^20^ The fluorescence lifetimes were longer (*τ* = 13.4 and 12.0 ns, respectively, Fig. S3) in comparison with [10]CPP (*τ* = 6.6 ns)^1*f*,21^ suggesting distinct excited-state dynamics influenced by pyrene integration and methoxy substitution patterns.

To investigate the influence of [2]OMe-Pyr-[8]CPP and [4]OMe-Pyr-[8]CPP on the carrier transport performance in OSCs, the molecules were respectively introduced into the D18:L8-BO-based OSCs. The chemical structures of D18 and L8-BO are shown in Fig. 1. The conventional structure of ITO/2PACz/active layer (100 nm)/PDINN (8.5 nm)/Ag (100 nm) was adopted to study the impact of introducing different molecules on the performance of OSCs.

**Fig. 4:**
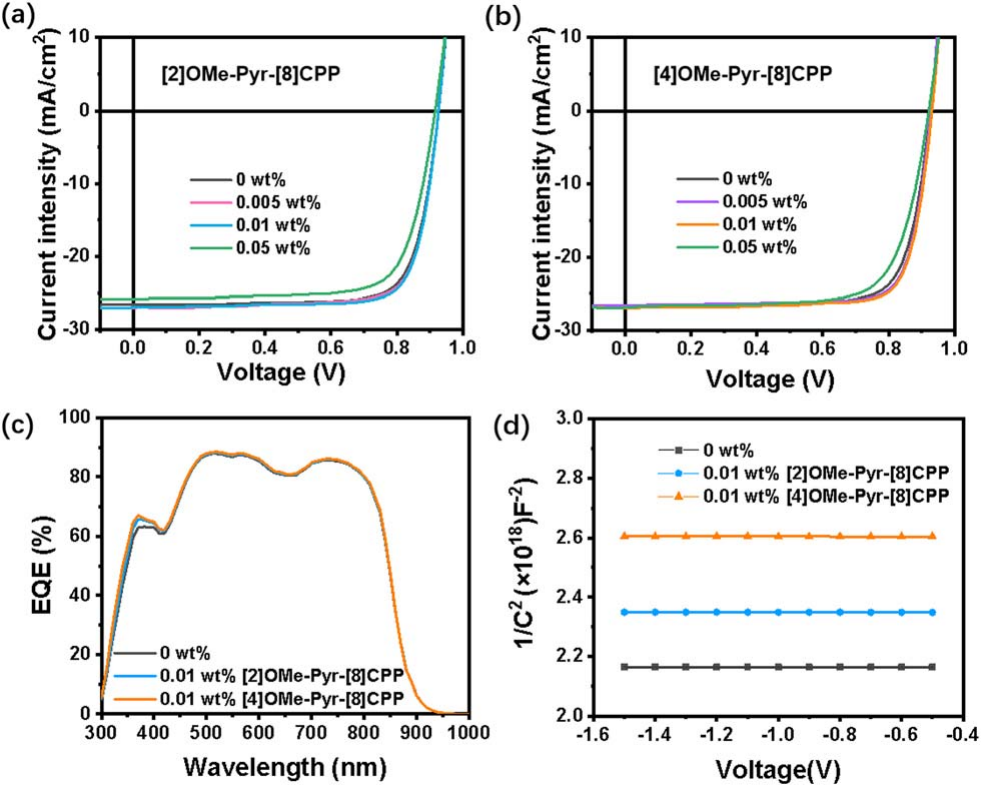
*J*–*V* plots of (a) [2]OMe-Pyr-[8]CPP and (b) [4]OMe-Pyr-[8]CPP, (c) EQE curves and (d) Mott–Schottky plot (1/*C*^2^–*V*) of 0.01 wt% [2]OMe-Pyr-[8]CPP and [4]OMe-Pyr-[8]CPP, respectively.

The current density–voltage (*J–V*) curves are shown in Fig. 4, and the corresponding photovoltaic parameters including short-circuit current (*J*_sc_), open-circuit voltage (*V*_oc_), fill factor (FF), and PCE are summarized in Table 1. The D18:L8-BO device exhibits a *V*_oc_ of 0.925 V, a *J*_sc_ of 26.55 mA cm^−2^, an FF of 77.62%, and a PCE of 19.24%. Upon incorporation of 0.005 wt% and 0.01 wt% dopant, the device performance is enhanced. Specifically, the incorporation of 0.01 wt% [2]OMe-Pyr-[8]CPP maintains the *V*_oc_ at 0.925 V, while achieving improved values of 26.93 mA cm^−2^ for *J*_sc_, 78.48% for FF, and a higher PCE of 19.73%. More notably, the introduction of [4]OMe-Pyr-[8]CPP results in a remarkable improvement in the photovoltaic performance of the D18:L8-BO system. The *V*_oc_ increases to 0.930 V, along with improvements in both *J*_sc_ (26.89 mA cm^−2^) and FF (79.11%). As a result, the PCE was significantly enhanced to 19.96%, ranking among the relatively high PCE values in doped OSC systems (see Fig. S4 and Table S3 in the SI). These result demonstrate that increasing the number of methoxy groups of the pyrene unit improves PCE, as evidenced by the comparison between [2]OMe-Pyr-[8]CPP and [4]OMe-Pyr-[8]CPP. However, when the doping concentration is increased to 0.05 wt% for both dopants [2]OMe-Pyr-[8]CPP and [4]OMe-Pyr-[8]CPP, a significant decline in PCE is observed (Fig. 4, S5, Table 1, S4 and SI). As evidenced by the data presented above, the incorporation of [n]OMe-Pyr-[8]CPP as a dopant at an optimal concentration can significantly enhance the device performance of OSCs. Moreover, a control experiment using [10]CPP as a dopant was performed to elucidate the unique contribution of the pyrene unit from the general effect of a macrocyclic structure. As illustrated in Fig. S6 and summarized in Table S5 (SI), devices doped with [10]CPP attained a PCE of only 18.64%, underperforming relative to those incorporating [2]OMe-Pyr-[8]CPP and [4]OMe-Pyr-[8]CPP at the same concentration. This marked difference unequivocally underscores the essential contribution of the pyrene unit beyond the general effect of the macrocyclic scaffold.

**Table 1:** Photovoltaic parameters of D18: L8-BO based OSCs with and without dopant

Device	*V*_oc_(V)	*J*_sc_/*J*_cal._^a^ (mA cm^−2^)	FF(%)	PCE_max_/PCE_ave_^b^ (%)
D18: L8-BO	0.925 (0.921 ± 0.005)	26.55 (25.15)	77.62 (76.43 ± 0.97)	19.24 (18.90 ± 0.35)
		(26.16 ± 0.48)		
0.005 wt% [2]OMe-Pyr-[8]CPP	0.925 (0.922 ± 0.004)	26.95 (25.15)	77.46 (76.51 ± 0.80)	19.48 (19.07 ± 0.28)
		(26.58 ± 0.38)		
0.01 wt% [2]OMe-Pyr-[8]CPP	0.925 (0.924 ± 0.004)	26.93 (25.28)	78.48 (77.51 ± 0.53)	19.73 (19.29 ± 0.33)
		(26.51 ± 0.32)		
0.05 wt% [2]OMe-Pyr-[8]CPP	0.925 (0.920 ± 0.004)	26.64 (22.67)	72.60 (72.11 ± 0.34)	17.61 (17.25 ± 0.36)
		(26.01 ± 0.55)		
0.005 wt% [4]OMe-Pyr-[8]CPP	0.927 (0.920 ± 0.004)	26.59 (25.21)	79.32 (78.31 ± 0.76)	19.72 (19.37 ± 0.37)
		(26.64 ± 0.22)		
0.01 wt% [4]OMe-Pyr-[8]CPP	0.930 (0.924 ± 0.003)	26.89 (25.37)	79.11 (78.41 ± 0.49)	19.96 (19.72 ± 0.23)
		(26.66 ± 0.28)		
0.05 wt% [4]OMe-Pyr-[8]CPP	0.921 (0.917 ± 0.003)	26.84 (23.82)	72.82 (72.29 ± 0.82)	17.97 (17.55 ± 0.33)
		(26.64 ± 0.37)		

a Calculated by integrating the product of the EQE with the AM1.5 G spectrum.

b Average data of 20 devices.

To elucidate the mechanism behind the enhancement of *V*_oc_, we supplemented the photoluminescence (PL) spectra of D18:L8-BO films with varying doping concentrations of [4]OMe-Pyr-[8]CPP. As shown in Fig. S7 (SI), a clear trend was observed: as the doping concentration of [4]OMe-Pyr-[8]CPP increases from 0 to 0.005 wt% and 0.01 wt%, the PL emission intensity gradually enhances. This result indicates that increasing CPP doping concentrations effectively passivates defects in the active layer and suppresses non-radiative recombination, thereby contributing to the enhancement of *V*_oc_ of devices.

To characterize the photoelectric response of OSCs, the external quantum efficiency (EQE) curves were recorded in the range of 300 to 1000 nm (Fig. S8, SI). As shown in Fig. 4c, according to the differential EQE curve between devices without dopants and with 0.01 wt% [n]OMe-Pyr-[8]CPPs, obvious increases in EQE value are observed in the wavelength range of 330 to 420 nm. This indicates that the EQE of the devices is enhanced after the introduction of [n]OMe-Pyr-[8]CPPs, thereby increasing the photocurrent. The integrated *J*_sc_ values from EQE spectra were 25.15, 25.28, and 25.37 mA cm^−2^ for devices without dopants, with [2]OMe-Pyr-[8]CPP, and with [4]OMe-Pyr-[8]CPP, respectively, closely aligning with the *J*_sc_ values obtained from the *J–V* characteristic curve under AM 1.5 G, 100 mW cm^−2^ illumination. In addition, the close agreement between the integrated EQE spectrum and the short-circuit current density from the *J*–*V* curve (∼5% deviation) verifies the accuracy of the *J*–*V* measurements for determining device performance. However, increasing the dopant concentration to 0.05 wt% leads to a reduction in EQE, consistent with the decline in overall device performance. These results indicate that the introduction of the [n]OMe-Pyr-[8]CPP dopant with optimized concentration can improve the photoelectric response of OSCs in a broad spectral region, thus facilitating improved *J*_sc_.

The change in carrier concentration (*n*) of the active layer after the addition of [2]OMe-Pyr-[8]CPP and [4]OMe-Pyr-[8]CPP can be obtained through capacitance–voltage (*C*–*V*) measurements. We fabricated electron-only devices with the structure of ITO/ZnO/D18: L8-BO (with or without 0.01 wt% [2]OMe-Pyr-[8]CPP and [4]OMe-Pyr-[8]CPP)/PDINN/Ag for *C*–*V* measurement analysis. The obtained *C*^2^–*V* curves are shown in Fig. 4d. The carrier concentration of the active layer with and without [n]OMe-Pyr-[8]CPPs is calculated according to Mott–Schottky analysis (see the SI), and summarized in Table 2. After adding 0.01 wt% [2]OMe-Pyr-[8]CPP, the *n* value of the active layer increases from 3.0 × 10^18^ cm^−3^ (control device without [n]OMe-Pyr-[8]CPPs) to 3.7 × 10^18^ cm^−3^. Notably, the optimized device containing [4]OMe-Pyr-[8]CPP achieves a substantially higher *n* value of 6.4 × 10^18^ cm^−3^, representing a 2.1-fold enhancement over the undoped control (no [n]OMe-Pyr-[8]CPPs). This increase demonstrates the remarkable efficacy of these pyrene-embedded nanohoops in boosting charge carrier density in the D18:L8-BO blend.

**Table 2:** Charge transport properties and carrier concentration of D18: L8-BO based OSCs with and without [n]OMe-Pyr-[8]CPPs (whose concentration is relative to D18: L8-BO)

Device	*µ*_h_ (10^−4^ cm^2^ V^−1^ s^−1^)	*µ*_e_ (10^−4^ cm^2^ V^−1^ s^−1^)	*µ*_h_/*µ*_e_	*n* (10^18^ cm^−3^)
D18: L8-BO	3.0	2.0	1.5	3.0
0.01 wt% [2]OMe-Pyr-[8]CPP	3.5	2.8	1.25	3.7
0.01 wt% [4]OMe-Pyr-[8]CPP	4.5	4.0	1.13	6.4

The carrier transport properties of the active layer were further investigated by the space-charge-limited-current (SCLC) method. The hole/electron mobility (*µ*_h_/*µ*_e_) of the devices was measured using the device structures of ITO/2PACz/active layer/MoO_3_/Ag (hole device) and ITO/ZnO/active layer/PDINN/Ag (electron device), respectively. The results are shown in Table 2. The incorporation of dopants leads to simultaneous enhancement in both hole and electron mobilities in the electronic devices. For hole mobility (*µ*_h_), the values increase progressively from 3.0 × 10^−4^ cm^2^ V^−1^ s^−1^(undoped device) to 3.5 × 10^−4^ cm^2^ V^−1^ s^−1^ ([2]OMe-Pyr-[8]CPP doped), and further to 4.5 × 10^−4^ cm^2^ V^−1^ s^−1^ ([4]OMe-Pyr-[8]CPP doped). Similarly, electron mobility (*µ*_e_) increases from 2.0 × 10^−4^ cm^2^ V^−1^ s^−1^ (undoped) to 2.8 × 10^−4^ cm^2^ V^−1^ s^−1^ ([2]OMe-Pyr-[8]CPP doped) and to 4.0 × 10^−4^ cm^2^ V^−1^ s^−1^ ([4]OMe-Pyr-[8]CPP doped). Notably, the *µ*_h_/*µ*_e_ ratio changes from 1.5 (undoped) to more balanced values of 1.25 ([2]OMe-Pyr-[8]CPP) and 1.13 ([4]OMe-Pyr-[8]CPP), demonstrating significantly improved charge transport balance upon dopant incorporation. This enhanced balance explains the superior photovoltaic performance of [4]OMe-Pyr-[8]CPP-doped OSCs. Mechanistically, the increased carrier concentration led to a reduction in the *µ*_h_/*µ*_e_ ratio while simultaneously suppressing carrier recombination, thereby improving both *J*_sc_ and FF of the OSCs.

To investigate the device stability, we conducted light stability tests on both doped and undoped devices with a structure of ITO/PEDOT:PSS/D18:L8-BO:[4]OMe-Pyr-[8]CPP/C_60_/BCP/Ag. As shown in Fig. S9 (SI), after 48 hours of continuous illumination, the doped device retained 61% of its initial PCE value and 93% of its initial current, whereas the control group (undoped one) retained only 13% and 19%, respectively.^22^ However, the voltage in both devices remained around 70% of their initial values. These results show that the introduction of [4]OMe-Pyr-[8]CPP does not compromise device stability due to electron transfer but instead significantly enhances photostability. We propose that the methoxy-pyrene-embedded CPPs, acting as n-type dopants, improve electron mobility and charge transport balance, thereby reducing localized electric field variations and material degradation caused by charge accumulation, which in turn mitigates power loss. Additionally, they may effectively fill charge traps in the active layer through the generated charges arising from charge transfer between the dopant and the acceptor, thus suppressing the formation of non-radiative recombination centers (causing energy loss) under prolonged illumination and thus slowing the decay of current density. As for the similar voltage decay rates in the two device groups, this indicates that the [4]OMe-Pyr-[8]CPP dopant does not significantly influence the bulk morphology of the active layer and the charge transport network.

To further probe the film morphology and molecular stacking behavior, grazing incidence wide-angle X-ray scattering (GIWAXS) measurements were carried out on D18:L8-BO films with 0 wt%, 0.005 wt%, and 0.01 wt% [4]OMe-Pyr-[8]CPP dopant. The 2D GIWAXS patterns (Fig. S10, SI) reveal no distinct changes in molecular packing or crystallinity between the undoped and doped devices, indicating that the dopant does not significantly affect the morphology. This observation aligns with the device stability results, collectively indicating that the performance enhancement in the doped devices is not related to morphological improvement.

**Fig. 5:**
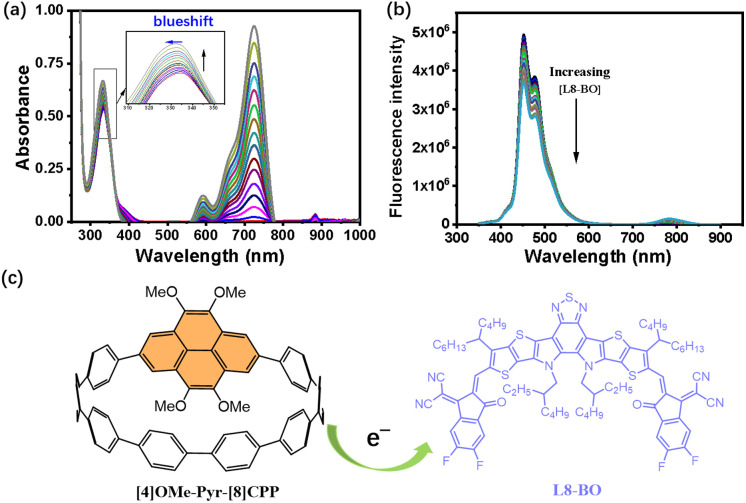
(a) Absorption spectral change upon titration of [4]OMe-Pyr-[8]CPP (conc. 2.5 × 10^−6^ M) with L8-BO (0–1.5 eq.) in CH_2_Cl_2_ at 298K. (b) Fluorescence emission spectral change upon titration of [4]OMe-Pyr-[8]CPP (conc. 5.0 × 10^−7^ M) with L8-BO (0–2.0 eq.) in CH_2_Cl_2_ at 298 K (*λ*_ex_ = 330 nm). (c) Schematic illustration of electron transfer from [4]OMe-Pyr-[8]CPP to L8-BO.

Based on the enhanced OSC performance, we propose that the electron-rich dopant facilitates intermolecular charge transport between the dopant and the L8-BO acceptor within the active layer, as evidenced by UV-vis and fluorescence emission titration spectra of [4]OMe-Pyr-[8]CPP with L8-BO in CH_2_Cl_2_ at 298 K (Fig. 5a). When L8-BO increases from 0 to 1.5 eq., the maximum absorption peak of [4]OMe-Pyr-[8]CPP blueshifts from 334 to 331 nm. Meanwhile, it can be observed that absorption intensity of [4]OMe-Pyr-[8]CPP gradually increases with the increase in L8-BO. While fluorescence titration reveals a gradual quenching of the [4]OMe-Pyr-[8]CPP emission intensity as the concentration of L8-BO increases (Fig. 5b). These spectroscopic changes unambiguously confirm charge transfer from the electron-rich nanohoop to the acceptor, corroborating the proposed dopant-mediated charge transport enhancement mechanism. These findings further highlight the essential function of the electron-rich, methoxy-substituted pyrene motif^23^ in promoting dopant–acceptor electronic communication, thereby contributing to the enhanced charge transport and device performance.

**Fig. 6:**
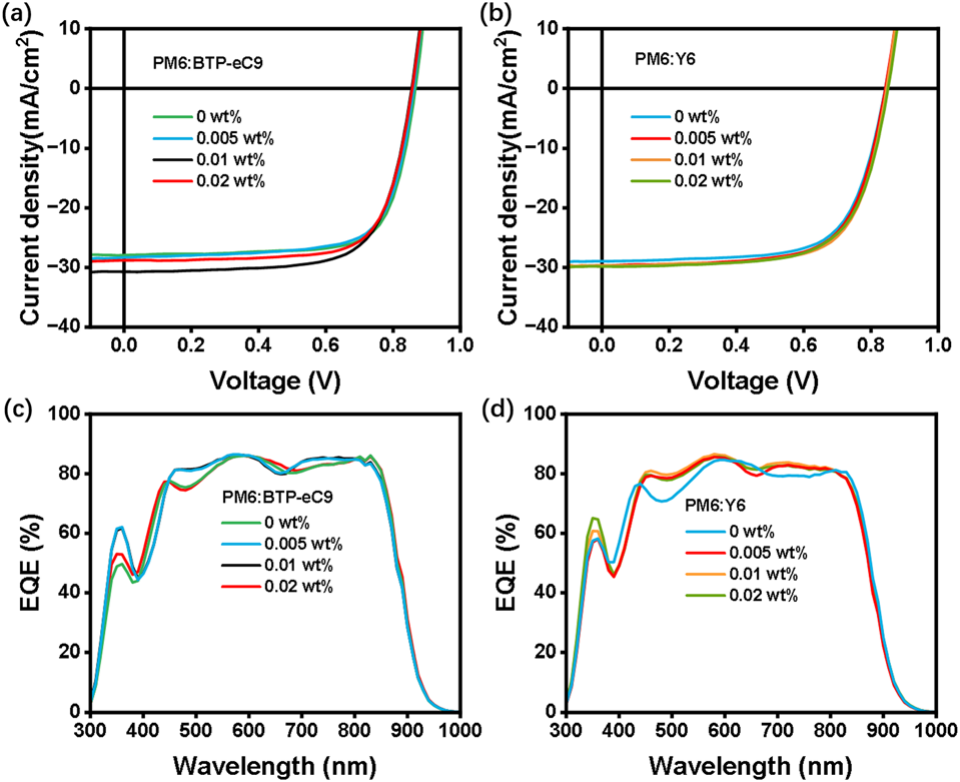
(a) *J*–*V* plots and (c) EQE curves of the PM6:BTP-eC9-based OSCs containing different concentrations of the [4]OMe-Pyr-[8]CPP dopant. (b) *J*–*V* plots and (d) EQE curves of the PM6:Y6-based OSCs containing different concentrations of the [4]OMe-Pyr-[8]CPP dopant.

To verify the universality of the dopant, we additionally selected two other D:A systems, PM6:BTP-eC9 and PM6:Y6, for experimentation. The chemical structures of PM6, BTP-eC9 and Y6 are shown in Fig. S12. The optimal parameters of the devices are shown in Table S6 and S7 (SI), and the *J–V* plots and corresponding EQE curves are shown in Fig. 6. The results show that after introducing the [4]OMe-Pyr-[8]CPP dopant, the *J*_sc_ of the devices is elevated relative to that of the control one. When using a proper dopant concentration (0.01 wt% for both PM6:BTP-eC9 and PM6:Y6 systems), an optimal PCE value can be obtained (18.44% and 17.51%). EQE spectra showed that devices based on PM6:BTP-eC9 and PM6:Y6 incorporating [4]OMe-Pyr-[8]CPP as a dopant exhibit good photoresponse in the range of 300–1000 nm, with notably enhanced EQE values in the 330–400 nm region compared to the control devices. The consistent performance enhancement across different systems demonstrates that the doping effect of CPPs possesses good universality.

## Conclusions

In conclusion, electron-rich pyrene-embedded CPP derivatives [n]OMe-Pyr-[8]CPPs have been successfully synthesized by incorporating pyrene groups into the CPP backbones. Single-crystal analysis unambiguously confirms their molecular structures, revealing a characteristic herringbone packing arrangement and the formation of extended tubular structures. Photophysical characterization reveals nearly identical UV-vis absorption and fluorescence emission profiles for both derivatives. Notably, when these nanohoops are introduced as n-type dopants into the D18:L8-BO active layer at an optimal concentration, the photovoltaic performance of OSCs is enhanced. Device physics measurements reveal that the addition of nanohoops elevates the carrier concentration and boosts hole and electron mobility. Consequently, the maximum PCE of doped devices increases from 19.24% to 19.96%. The enhanced performance of the OSCs can be attributed to the electron-rich dopant promoting efficient intermolecular charge transport between the dopant and L8-BO acceptor. This work expands the scope of the synthesis of functional nanohoops based on curved π-conjugated molecules and paves the way for the application of CPP derivatives in OSC devices.

## Author contributions

J. H. synthesized and characterized all materials, and drafted the manuscript. W. L. fabricated and evaluated the OSCs, and contributed to manuscript drafting. Q. X. performed additional data analysis related to OSCs. S. W., Z. L., X. L. and S. G assisted with partial synthesis. Y. W. carried out DFT calculations and simulated photophysical spectra. X. X. reviewed and refined the manuscript. H. J. supervised the project, revised the manuscript, and acquired funding. All authors have given approval to the final version of the manuscript.

## Conflicts of interest

There are no conflicts to declare.

## Supplementary Material

SC-OLF-D5SC06584D-s001

SC-OLF-D5SC06584D-s002

## Data Availability

CCDC 2414163 ([2]OMe-Pyr-[8]CPP) and 2414429 ([4]OMe-Pyr-[8]CPP) contain the supplementary crystallographic data for this paper.^18*a*,*b*^ The data that support the findings of this study are available in the supplementary information (SI) of this article. Supplementary information is available. See DOI: https://doi.org/10.1039/d5sc06584d.
