# Supplementary material for: Pyrene-embedded nanohoops: synthesis and dopant engineering for organic solar cells with an enhanced efficiency of 19.96%
Source: Chem Sci. 2026 Jan 13;17(10):5192–200. doi: 10.1039/d5sc06584d (PMC12815244; doi:10.1039/d5sc06584d)
Supplement: SC-017-D5SC06584D-s001 [file SC-017-D5SC06584D-s001.pdf]

Supplementary Information (SI) for Chemical Science.
This journal is © The Royal Society of Chemistry 2026

Supporting Information

Pyrene-Embedded Nanohoops: Synthesis and Dopant Engineering for Organic Solar Cells with Enhanced Efficiency of 19.96%

Jing He,[+] Wenlong Liu,[+] Siwei Wu, Qi Xie, Zhe Lian, Xiaonan Li, Shengzhu Guo, Ying Wang,* Xinjun Xu,* and Hua Jiang*

College of Chemistry, Beijing Normal University, Beijing 100875 (P.R. China)

Table of Contents

1. Experimental Procedures 3
1.1 General Procedures and Materials 3
1.2 Device Fabrication 3
1.3 *J*-*V* and EQE measurements 4
1.4 Carrier concentration calculation 4
1.5 SCLC mobility measurements 4
1.6 GIWAXS measurements 4
1.7 Synthetic Procedures 4
2. Results and Discussion 7
2.1 X-ray Crystallography 7
2.2 Photophysical Properties 10
2.3 Comparison of PCE values for some reported dopants in OSCs 11
2.4 Device performance of OSCs doped with 0.05 wt% [n]OMe-Pyr-[8]CPP 13
2.5 OSC doped with [10]CPP 13
2.6 PL emission of the active layer 14
2.7 EQE curves of [n]OMe-Pyr-[8]CPP 14
2.8 The stability performance of OSCs 15
2.9 GIWAXS images 15
2.10 UV-vis spectra of L8-BO 16
2.11 The universality of the dopant 17
2.12 Computational details and results 18
2.13 ^{1}H, ^{13}C NMR and Mass Spectra 31
References 35

1. Experimental Procedures

1.1 General Procedures and Materials

All starting chemicals were obtained from commercial sources and used without further purification, unless indicated otherwise. Per-deuterated solvents for NMR spectroscopy were obtained from Cambridge Isotope Laboratories. All reactions were performed with dry solvents under Argon in dried glassware with standard vacuumline techniques. Anhydrous 1,4-dioxane and THF were obtained from Solvent Purification System. Compound **1**,[S1] ***2***,[S2] **3**,[S3] **4**[S3] were prepared according to the literatures. Column chromatography was carried out on flash grade silica gel, using 0 - 20 psig pressure. Analytical TLC was carried out using tapered silica plates with a preadsorbent zone. Nuclear magnetic resonance (NMR) spectra were obtained with JEOL Delta (400 MHz and 600 MHz) using chloroform-*d* ($CDCl_3$) as solvent. The chemical shift references were as follows: (^{1}H) chloroform-*d*, 7.26 ppm; (^{13}C) chloroform-*d*, 77.00 ppm (chloroform-*d*). Multiplicities are abbreviated as follows: s = singlet, d = doublet, t = triplet, m = multiplet, and br = broad. Coupling constants (*J*) are given in hertz (Hz). Mass spectra (ESI, MALDI) were acquired on GCT and FTICR spectrometer (Bruker Daltonics Inc. APEXII, BIFLEX III), respectively. Single crystal X-ray diffraction data were collected on a Rigaku Super Nova, Dual, Cu at zero, AtlasS2 diffractometer. Fluorescence spectra were measured on FS5 and FLS980, and UV−Vis spectra were recorded on Shimadzu UV−3600. GIWAXS measurements were performed at 1W1A Diffuse X-ray Scattering Station, Beijing Synchrotron Radiation Facility (BSRF-1W1A).

1.2 Device Fabrication

The photovoltaic performance of the D18:L8-BO based OSCs with the structure of indium tin oxide (ITO)/[2-(9H-Carbazol-9-yl)ethyl]phosphonic acid (2PACz)/active layer (100 nm)/N,N'-bis[3-[3-(dimethylamino)propylamino]propyl]perylene-3,4:9,10-bis(dicarboximide) (PDINN) (8.5 nm)/Ag (100 nm) was characterized. The pre-cleaned ITO substrates were treated with UVO-ozone for 20 minutes. 2PACz (0.3 mg mL^{-1} in anhydrous ethanol) was spin-coated at 4000 rpm for 20 s and then annealed at 80°C for 3 minutes. After that, it was transferred to the glove box. Different concentrations of chloroform dissolved dopants (the optimal concentration was 0.01 wt% for the acceptor) were prepared. The active layer of D18:L8-BO = 1:1.2 (5 mg mL^{-1}) containing the dopants was dissolved by heating for 1 hour and then cooled to 85°C, followed by spin-coating at 2500 rpm for 20 s (about 100 nm). Immediately after spin-coating, it was thermally annealed on a hot stage at 100°C for 60 s. Subsequently, PDINN (1.5 mg ml^{-1} in methanol) was spin-coated at 3000 rpm for 30 s on the active layer. Finally, a 100 nm silver layer was deposited in an ordered manner under a vacuum pressure of 10^{-7} Torr. The effective area of the device was 0.04 cm^2.

PM6:BTP-eC9 and PM6:Y6 based devices adopt the structure of ITO/poly (3, 4- ethylenedioxythiophene): poly(styrenesulfonate) (PEDOT:PSS)/active layer (100 nm)/PDINN (8.5 nm)/Ag (100 nm). Pre-cleaned ITO substrates were first treated with UV-ozone for 25 min. Aqueous PEDOT:PSS solution (diluted with deionized water in a 1:1 volume ratio) was then spin-coated at 3500 rpm for 20 s, followed by annealing at 150 °C for 12 min. The substrates were subsequently transferred into a nitrogen-filled glovebox. The active layer was prepared from chloroform solutions containing different dopant concentrations (0.005, 0.01, 0.02 wt%; the optimal doping concentration was determined to be 0.01 wt% relative to the acceptor). The donor-to-acceptor weight ratio was maintained at PM6:BTP-eC9 (or PM6:Y6) = 1:1.2, with a total concentration of 15.8 mg mL^{-1}. The blend solution was stirred at an elevated temperature for 1.5 h and cooled to room temperature before being spin-coated at 2350 rpm for 20 s to yield a film of approximately 100 nm. Immediately after deposition, the active layer was thermally annealed at 90 °C for 5 min. A PDINN interlayer (1.5 mg mL^{-1} in methanol) was then spin-coated at 3000 rpm for 30 s. Finally, a 100 nm thick Ag electrode was thermally evaporated under a vacuum of ~10^{-7} Torr. The active area of the device was defined as 0.04 cm^2.

1.3 *J-V* and EQE measurements

The current density-voltage (*J–V*) characteristics of the OSCs were recorded with a Keithley 2450. The power conversion efficiencies (PCEs) of the OSCs were measured under 1 sun, AM 1.5G (air mass 1.5 global) (100 mW cm^{-2}), using a SS-F5-3A (Enli Technology CO., Ltd.) solar simulator (AAA grade, 50 mm × 50 mm photo-beam size) of Enli Technology Co., Ltd. (China). 2 × 2 cm^2 monocrystalline silicon reference cell (SRC-00019, covered with a KG5 filter windows) was purchased from Enli Technology Co., Ltd. The active areas were determined to be 0.0260 cm^2 by masks. The external quantum efficiency (EQE) was measured by Solar Cell Spectral Response Measurement System (QE-R3011, Enli Technology Co., Ltd.). The light intensity at each wavelength was calibrated with a standard single-crystal Si photovoltaic cell.

1.4 Carrier concentration calculation

The change in carrier concentration of the active layer after the addition of **[2]OMe-Pyr-[8]CPP** and **[4]OMe-Pyr-[8]CPP** can be obtained through *C–V* measurements. We fabricated pure electron devices with the structure of ITO/ZnO/D18: L8-BO (with or without **[2]OMe-Pyr-[8]CPP** and **[4]OMe-Pyr-[8]CPP**)/PDINN/Ag for *C–V* measurement analysis. The obtained $1/C^2$–V curves are shown in the figure. The formula for calculating the carrier concentration is:

$$\frac{\partial C^{-2}}{\partial V} = \frac{2}{qnA^2\varepsilon_r\varepsilon_0} \qquad (1)$$

where C is the capacitance, V is the voltage, ε_0 is the vacuum permittivity, ε_r is the relative permittivity, q is the elementary charge, n is the carrier concentration, and A is the surface area of the solar cell.

1.5 SCLC mobility measurements

The hole and electron mobilities of devices were evaluated from the space-charge limited current (SCLC) method with the hole-only structure of ITO/2PACz/active layer/MoO_3/Ag and electron-only structure of ITO/ZnO/active layer/PDINN/Ag, respectively. The corresponding charge mobilities were calculated from fitting the Mott-Gurney square law:

$$J = \frac{9\varepsilon_r\varepsilon_0\mu V^2}{8d^3} \qquad (2)$$

where J is the current density, ε_r is the dielectric permittivity of the active layer (assumed to be 3), ε_0 is the vacuum permittivity, d is the thickness of the active layer, and μ is the hole or electron mobility. $V = V_{appl}–V_{bi}$, V_{appl} is the applied voltage, V_{bi} is the built-in voltage. The processing conditions used for the charge mobility measurements are the same as that in the optimal OSCs. Tin foil was used to protect the devices from light during measurement. The SCLC devices were measured under a dark condition in a nitrogen glovebox without encapsulation.

1.6 GIWAXS measurements

GIWAXS measurements for the D18:L8-BO blend films were conducted at 1W1A Diffuse X-ray Scattering Station, Beijing Synchrotron Radiation Facility (BSRF-1W1A). The samples were prepared on silicon substrates using the same blend solutions as those used for device fabrication. A 10 keV X-ray beam was used with a grazing incidence angle of 0.2°, selected to ensure the film signals were effectively probed. LaB_6 was used as a standard for calibration prior to measuring the blend films. During data processing, the GIWAXS patterns were normalized for both exposure time and film thickness.

1.7 Synthetic Procedures

Compounds 1a[S1], 1b[S1], 1[S1], 2a[S2], 2b[S2], 2[S2], 3a[S3], 3b[S3], 3c[S3], 3d[S3], 3e[S3], 3[S3] and 4[S3] were synthesized following the procedures described in the corresponding literature. Their ^{1}H NMR spectra were consistent with the reported data.

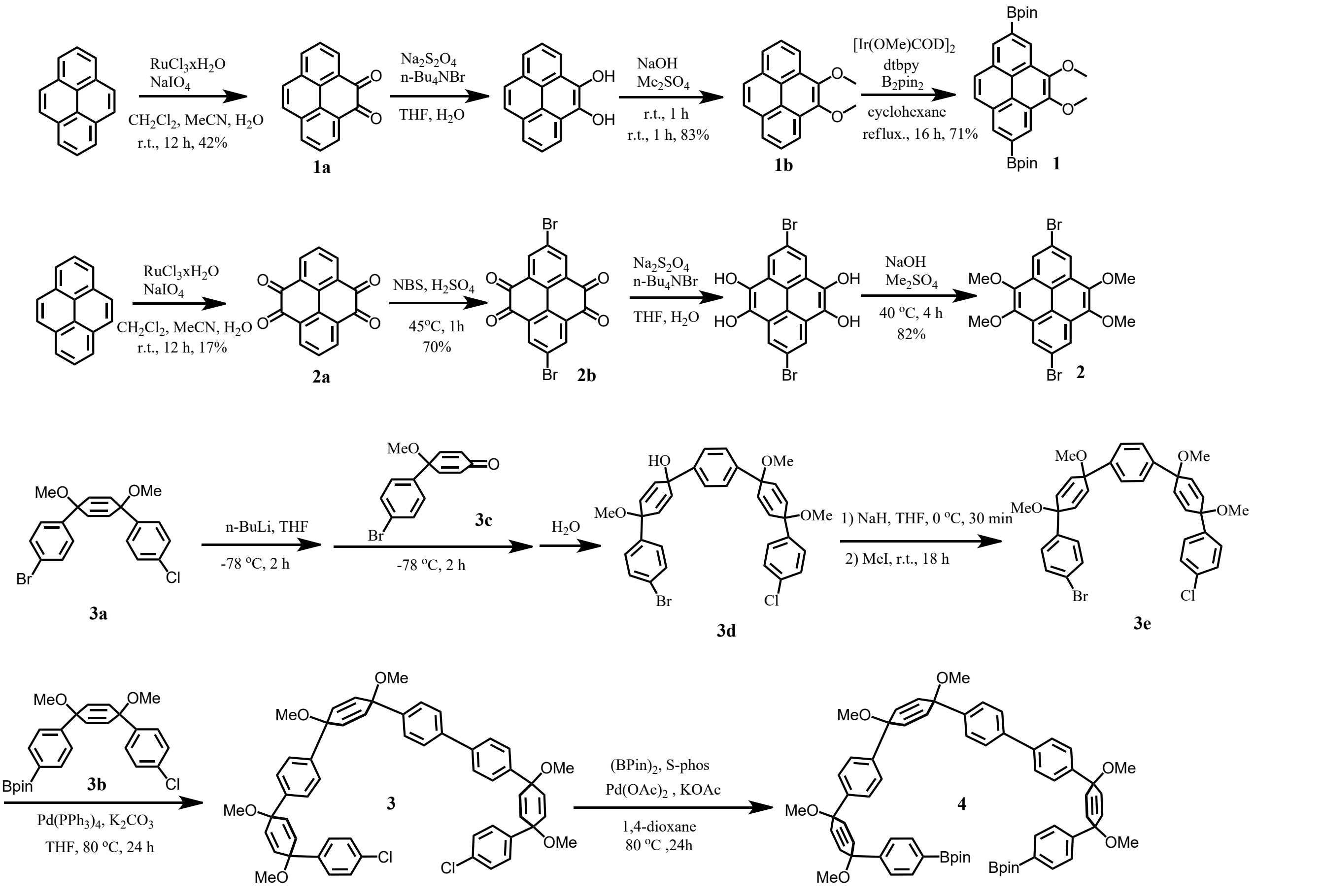

General synthesis method and characterization of [2]OMe-Pyr-[8]CPP

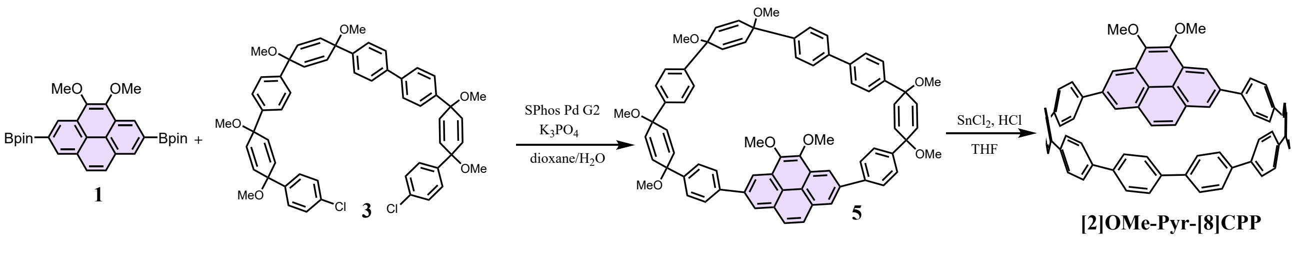

To a dry two-necked flask charged with a stir bar was added **1**:2,7-bis-(Bpin)-4,5-dimethoxypyrene (311 mg, 0.605 mmol), **3** (476 mg, 0.550 mmol), SPhos Pd G2 (159 mg, 0.221 mmol). The flask was fitted with a rubber septum and evacuated/backfilled with N_2 (3x) before the addition of anhydrous dioxane (150 mL) under an N_2 atmosphere. The reaction mixture was heated to 80 °C for ca. 10 min whereupon degassed (sparged 1 h) 2 M aq. K_3PO_4 (30 mL) was added via cannula. The resulting dark yellow solution was heated under N_2 for 48 h. The crude was diluted with EA (100 mL) and washed with water (80 mL × 3) and brine (80 mL). The organic layer was dried over anhydrous Na_2SO_4 and the solvent was removed under reduced pressure afford crude product **5** which was used on the next step without further purification.

A H_2SnCl_4/THF solution was freshly prepared by dissolving anhydrous $SnCl_2$ (4.21 g, 22.2 mmol) in 34.0 mL anhydrous THF under nitrogen atmosphere and then adding concentrated HCl (aq.) (3.7 mL) to the solution. The resulting solution was deoxygenated and stirred for 15 minutes before use. The above crude product was dissolved in anhydrous THF (30 mL) under a nitrogen atmosphere and the freshly prepared H_2SnCl_4/THF solution (37 mL) was added to this solution. The reaction mixture was stirred at room temperature for 16 h before being quenched with NaOH/H_2O solution. The aqueous layer was extracted with dichloromethane and the organic layers were combined and dried with anhydrous Na_2SO_4. The crude product was purified by column chromatography on silica gel (DCM/PE, 1/1, v/v) to get **[2]OMe-Pyr-[8]CPP** a yellow-greenish solid (143 mg, 30 % yield, over two steps). ^{1}H NMR (600 MHz, Chloroform-*d*, 298K) δ = 8.50 (s, 2H), 8.17 (s, 2H), 7.90 (s, 2H), 7.74 (d, J = 8.6 Hz, 4H), 7.66-7.61 (m, 28H), 4.19 (d, J = 1.8 Hz, 6H). ^{13}C NMR (150 MHz, Chloroform-*d*, 298K) δ = 144.5, 138.7, 138.3, 138.2, 138.1, 138.1, 138.0, 137.9, 137.9, 137.2, 131.8, 129.2, 128.1, 127.6, 127.5, 127.3, 127.3, 127.2, 127.2, 123.4, 122.1, 118.9, 61.4. HRMS (MALDL-TOF) calculated for $C_{66}H_{45}O_2$ $[M+H]^+$: 869.3414, found 869.3402.

General synthesis method and characterization of [4]OMe-Pyr-[8]CPP

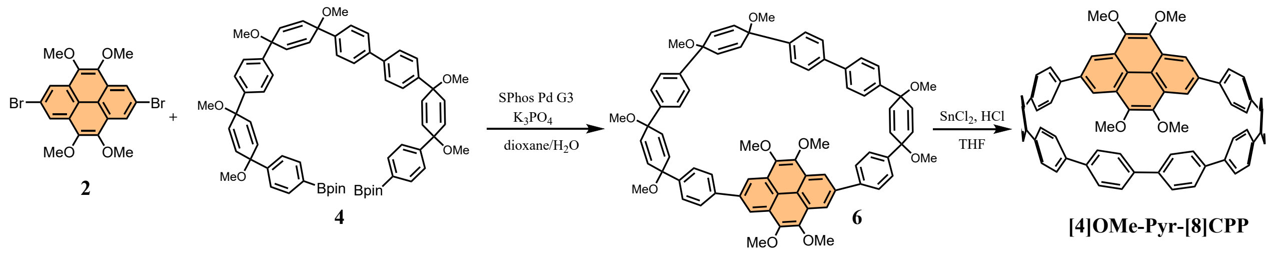

[4]OMe-Pyr-[8]CPP

To a dry two-necked flask charged with a stir bar was added **2**: 2,7-dibromo-4,5,9,10-tetramethoxypyrene (200 mg, 0.416 mmol), **4** (480 mg, 0.458 mmol), SPhos Pd G3 (130 mg, 0.166 mmol). The flask was fitted with a rubber septum and evacuated/backfilled with N_2 (3x) before the addition of anhydrous dioxane (160 mL) under an N_2 atmosphere. The reaction mixture was heated to 80 ^{o}C for ca. 10 min whereupon degassed (sparged 1 h) 2 M aq. K_3PO_4 (32 mL) was added via cannula. The resulting dark yellow solution was heated under N_2 for 48 h. The crude was diluted with EA (100 mL) and washed with water (80 mL × 3) and brine (80 mL). The organic layer was dried over anhydrous Na_2SO_4 and the solvent was removed under reduced pressure afford crude product **6** which was used on the next step without further purification.

A H_2SnCl_4/THF solution was freshly prepared by dissolving anhydrous $SnCl_2$ (2.81 g, 14.8 mmol) in 22.4 mL anhydrous THF under nitrogen atmosphere and then adding concentrated HCl (aq.) (2.5 mL) to the solution. The resulting solution was deoxygenated and stirred for 15 minutes before use. The above crude product was dissolved in anhydrous THF (20 mL) under a nitrogen atmosphere and the freshly prepared H_2SnCl_4/THF solution (25 mL) was added to this solution. The reaction mixture was stirred at room temperature for 16 h before being quenched with NaOH/H_2O solution. The aqueous layer was extracted with dichloromethane and the organic layers were combined and dried with anhydrous Na_2SO_4. The crude product was purified by column chromatography on silica gel (DCM/PE, 1/1, v/v) to get **[4]OMe-Pyr-[8]CPP** a yellow-greenish solid (104 mg, 27 % yield, over two steps). ^{1}H NMR (600 MHz, Chloroform-*d*) δ 8.46 (s, 4H), 7.77 (d, J = 8.7 Hz, 4H), 7.58-7.51 (m, 28H), 4.18 (s, 12H). ^{13}C NMR (150 MHz, Chloroform-*d*) δ 144.4, 138.8, 138.3, 138.2, 138.1, 138.0, 137.9, 137.6, 129.2, 128.2, 127.6, 127.5, 127.3, 127.3, 127.2, 127.2, 120.0, 118.3, 61.47. HRMS (MALDL-TOF) calculated for $C_{68}H_{49}O_4$ $[M+H]^+$: 929.3625, found 929.3620.

2. Results and Discussion

2.1 X-ray Crystallography

Crystals suitable for X-ray analysis were obtained by vapor diffusion of hexane into dichloromethane solution of **[2]OMe-Pyr-[8]CPP** and **[4]OMe-Pyr-[8]CPP**, respectively. Single crystal X-ray diffraction data were collected on a Rigaku Super Nova, Dual, Cu at zero, AtlasS2 diffractometer. The crystal was kept at 100.00(10) K during data collection. Using Olex2[S4], the structure was solved with the ShelXT[S5] structure solution program using Direct Methods and refined with the ShelXL[S6] refinement package using Least Squares minimization. The disordered solvent molecules were removed with the SQUEEZE routine in PLATON[S7] and the solvent-free model was employed for the final refinement. All non-hydrogen atoms were refined anisotropically. All hydrogen atoms were positioned by geometric idealization. Details of the crystal data and a summary of the intensity data collection parametersare listed in Table S4-S5. Crystallographic data were deposited at the Cambridge Crystallographic Data Center (CCDC 2414163 for **[2]OMe-Pyr-[8]CPP**, CCDC 2414429 for **[4]OMe-Pyr-[8]CPP**). The data can be obtained free of charge from The Cambridge Crystallographic Data Centre via www.ccdc.cam.ac.uk/structures.

Crystal Structure Data of Compound [2]OMe-Pyr-[8]CPP (CCDC number: 2414163).

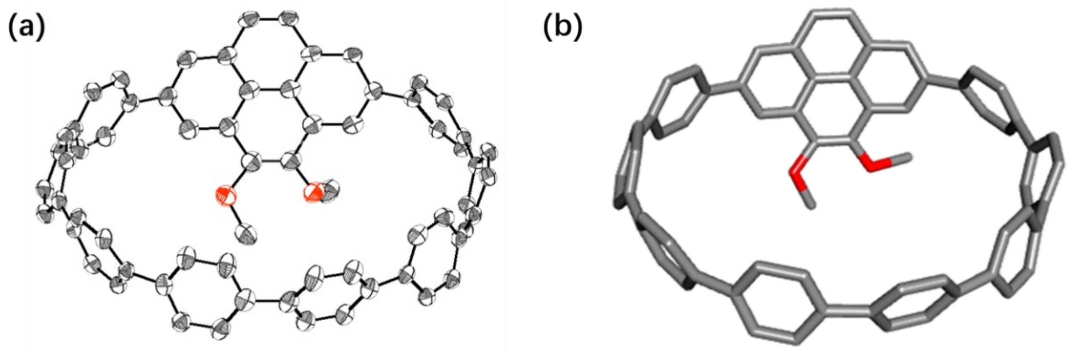

Figure S1. Crystal structure of **[2]OMe-Pyr-[8]CPP** was obtained by slow diffusion of hexane into dichloromethane solution. (a) ORTEP drawing (b) Crystal structure. Hydrogen atoms are omitted for clarity. Thermal ellipsoids are drawn at the 50% probability level.

Table S1. Crystal data and structure refinement for compound **[2]OMe-Pyr-[8]CPP**.

	**[2]OMe-Pyr-[8]CPP**
CCDC	2414163
Empirical formula	$C_{68}H_{48}Cl_4O_2$
Formula weight	1038.86
Temperature/K	100.00(10)
Crystal system	monoclinic
Space group	Pc
a/Å	17.3171(4)
b/Å	9.62150(15)
c/Å	16.4770(4)
α/°	90
β/°	109.052(2)
γ/°	90

Volume/Å^3	2594.97(10)
Z	2
ρ_{calc}g/cm^3	1.330
μ/mm^{-1}	2.444
F(000)	1080.0
Crystal size/mm^3	0.35 × 0.3 × 0.3
Radiation	Cu Kα (λ = 1.54184)
2Θ range for data collection/°	9.192 to 152.198
Index ranges	-21 ≤ h ≤ 21, -11 ≤ k ≤ 8, -20 ≤ l ≤ 18
Reflections collected	16635
Independent reflections	8049 [R_{int} = 0.0357, R_{sigma} = 0.0388]
Data/restraints/parameters	8049/297/764
Goodness-of-fit on F^2	1.060
Final R indexes [I>=2σ (I)]	R_1 = 0.0786, wR_2 = 0.2152
Final R indexes [all data]	R_1 = 0.0836, wR_2 = 0.2220
Largest diff. peak/hole / e Å^{-3}	1.06/-0.66

Crystal Structure Data of Compound [4]OMe-Pyr-[8]CPP (CCDC number: 2414429).

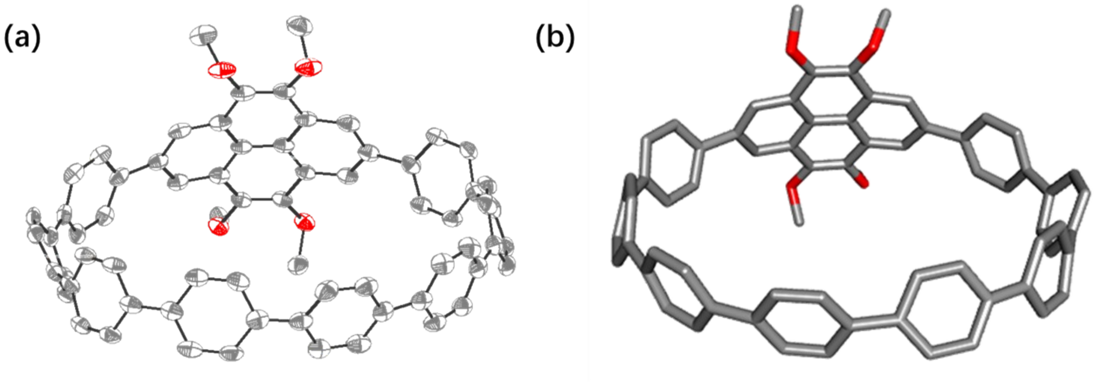

Figure S2. Crystal structure of **[4]OMe-Pyr-[8]CPP** was obtained by slow diffusion of hexane into chloroform solution. (a) ORTEP drawing (b) Crystal structure. Hydrogen atoms are omitted for clarity. Thermal ellipsoids are drawn at the 50% probability level.

Table S2. Crystal data and structure refinement for compound **[4]OMe-Pyr-[8]CPP**.

	**[4]OMe-Pyr-[8]CPP**
CCDC	2414429
Empirical formula	$C_{69}H_{50}Cl_2O_4$
Formula weight	1013.99
Temperature/K	100.00(10)
Crystal system	monoclinic
Space group	Cc
a/Å	33.5055(11)
b/Å	10.0230(3)
c/Å	14.7840(4)
α/°	90
β/°	96.225(3)
γ/°	90
Volume/Å^3	4935.6(3)

Z	4
ρ_{calc}g/cm^3	1.365
μ/mm^{-1}	1.615
F(000)	2120.0
Crystal size/mm^3	0.35 × 0.3 × 0.3
Radiation	Cu Kα (λ = 1.54184)
2Θ range for data collection/°	9.214 to 134.16
Index ranges	$-40 \leq h \leq 39$, $-11 \leq k \leq 10$, $-17 \leq l \leq 17$
Reflections collected	15406
Independent reflections	6803 [R_{int} = 0.0612, R_{sigma} = 0.0705]
Data/restraints/parameters	6803/64/708
Goodness-of-fit on F^2	1.042
Final R indexes [I>=2σ (I)]	R_1 = 0.0639, wR_2 = 0.1658
Final R indexes [all data]	R_1 = 0.0693, wR_2 = 0.1721
Largest diff. peak/hole / e Å^{-3}	0.41/-0.37

2.2 Photophysical Properties

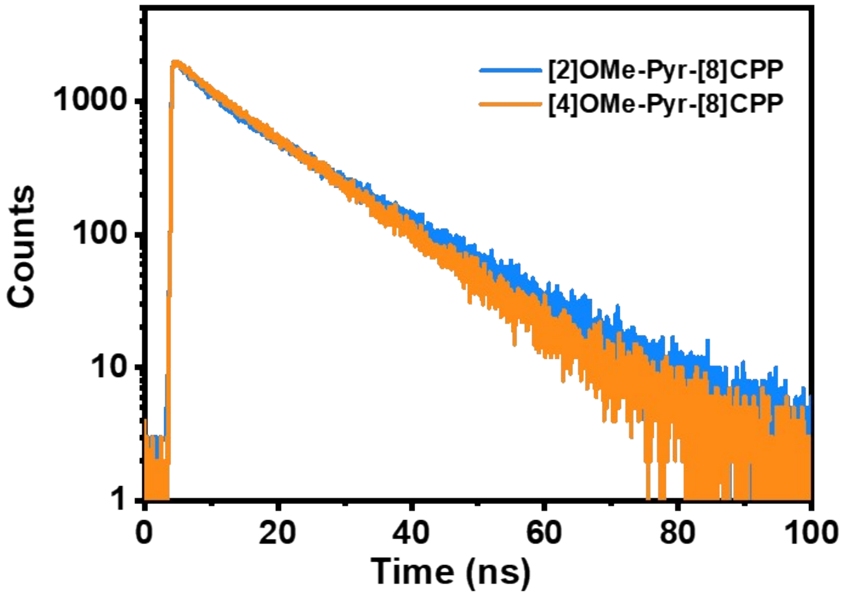

Figure S3. Emission lifetime of **[2]OMe-Pyr-[8]CPP** and **[4]OMe-Pyr-[8]CPP** in dichloromethane ($c = 1.0 \times 10^{-5}$ M).

2.3 Comparison of PCE values for some reported dopants in OSCs

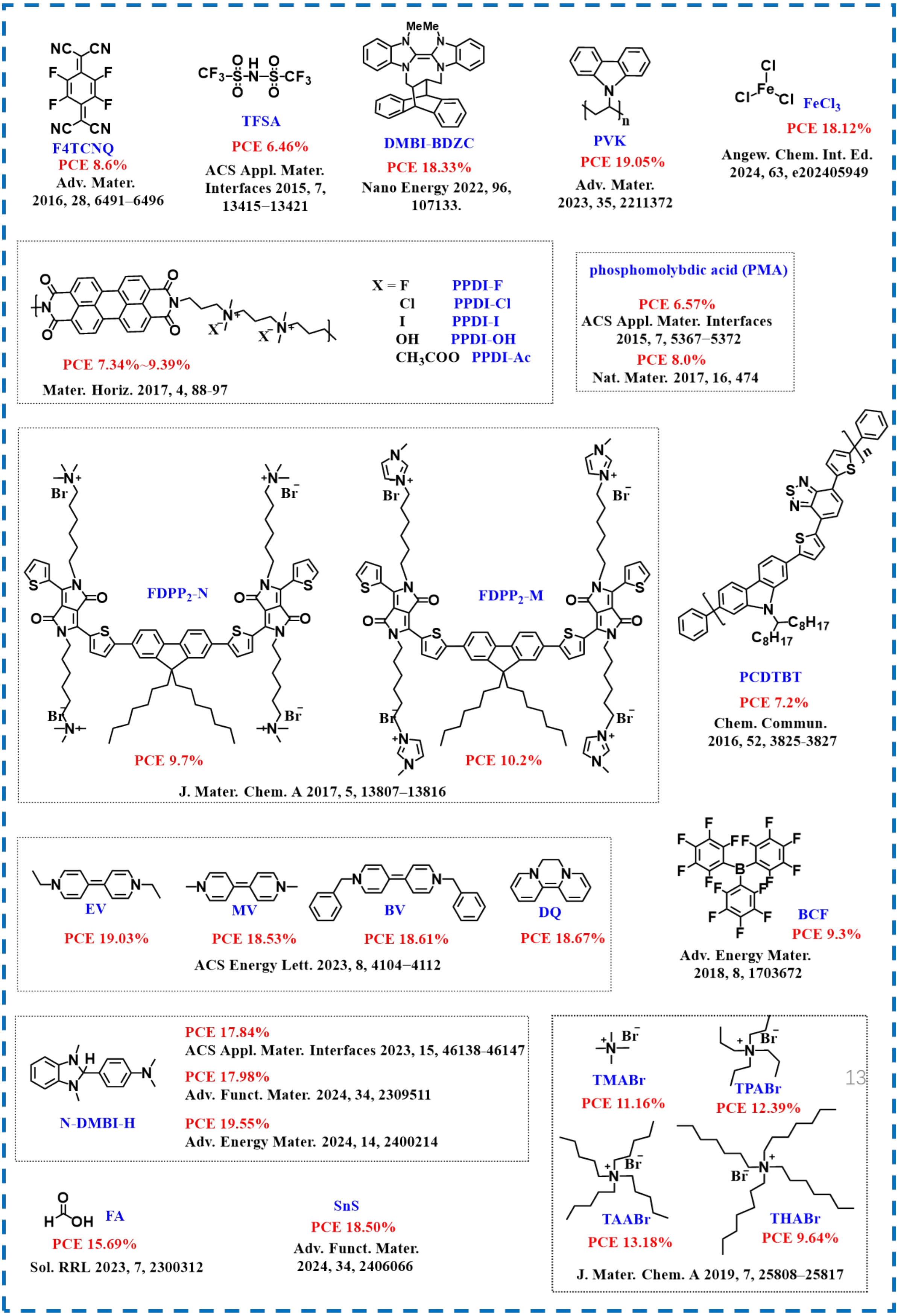

Figure S4. Some reported dopants for OSCs.

Table S3. Summary of PCE values for some reported dopants in OSCs.

Compounds	PCE（%）	Ref.
**F4TCNQ**	8.6	*Adv. Mater.* **2016**, *28,* 6491-6496.
**TFSA**	6.46	*ACS Appl. Mater. Interfaces* **2015**, *7,* 13415-13421.
**DMBI-BDZC**	18.33	*Nano Energy* ***2022***, *96,* 107133.
**PVK**	19.05	*Adv. Mater.* ***2023***, *35,* 2211372.
**$FeCl_3$**	18.12	*Angew. Chem. Int. Ed.* **2024**, *63,* e202405949.
**PPDI-X, X= F, Cl, I, OH, Ac**	7.34~9.39	*Mater. Horiz.* **2017**, *4,* 88-97.
**PMA**	6.57	*ACS Appl. Mater. Interfaces* **2015**, *7,* 5367-5372.
**PMA**	8.0	*Nat. Mater.* **2017**, *16,* 474.
**SnS**	18.5	*Adv. Funct. Mater.* **2024**, *34,* 2406066.
**$FDPP_2$-N**	9.7	*J. Mater. Chem. A* **2017**, *5,* 13807-13816.
**$FDPP_2$-M**	10.2	
**PCDTBT**	7.2	*Chem. Commun.* **2016**, *52,* 3825-3827.
**EV**	19.03	*ACS Energy Lett.* **2023**, *8,* 4104-4112.
**MV**	18.53	
**BV**	18.61	
**DQ**	18.67	
**BCF**	9.3	*Adv. Energy Mater.* **2018**, *8,* 1703672.
**N-DMBI-H**	17.84	*ACS Appl. Mater. Interfaces* **2023**, *15,* 46138-46147.
**N-DMBI-H**	17.98	*Adv. Funct. Mater.* **2024**, *34,* 2309511.
**N-DMBI-H**	19.55	*Adv. Energy Mater.* **2024**, *14,* 2400214.
**FA**	15.69	*Sol. RRL* **2023**, *7,* 2300312.
**SnS**	18.5	*Adv. Funct. Mater.* **2024**, *34,* 2406066.
**TMABr**	11.16	*J. Mater. Chem. A,* **2019**, *7,* 25808-25817.
**TPABr**	12.39	
**TAABr**	13.18	
**THABr**	9.64	

2.4 Device performance of OSCs doped with 0.05 wt% [n]OMe-Pyr-[8]CPP

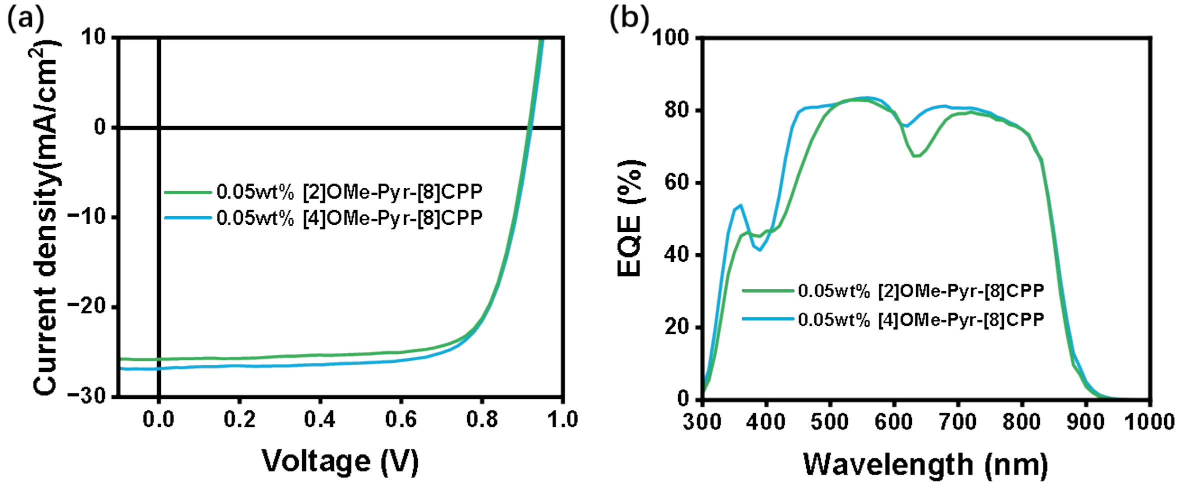

Figure S5. (a) *J–V* plots and (b) EQE curves of OSCs doped with 0.05 wt% **[2]OMe-Pyr-[8]CPP** and 0.05 wt% **[4]OMe-Pyr-[8]CPP**.

Table S4. Photovoltaic parameters of D18: L8-BO based OSCs doped with 0.05 wt% **[n]OMe-Pyr-[8]CPP**.

Device	V_{oc} (V)	J_{sc}/$J_{cal.}$ (mA cm^{-2})	FF (%)	PCE$_{max}$/PCE$_{ave}$ (%)
0.05wt% **[2]OMe-Pyr-[8]CPP**	0.920	26.01 (22.67)	72.11	17.61/17.25
0.05wt% **[4]OMe-Pyr-[8]CPP**	0.917	26.21 (23.82)	71.69	17.97/17.55

2.5 OSC doped with [10]CPP

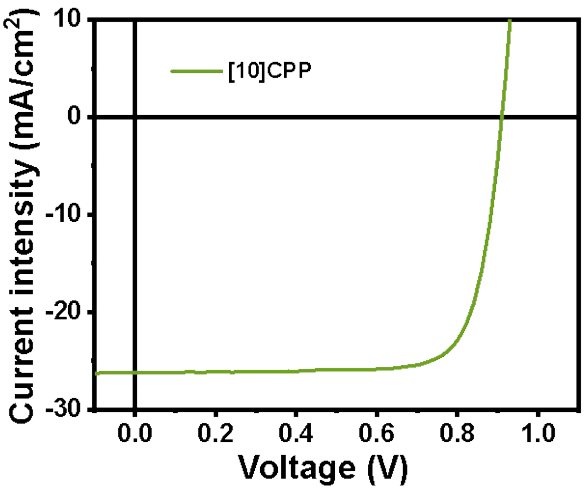

Figure S6. *J-V* plots of D18: L8-BO based OSCs doped with 0.01 wt% **[10]CPP**.

Table S5. Photovoltaic parameters of D18: L8-BO based OSCs doped with 0.01 wt% **[10]CPP**.

D:A	Dopant	V_{oc} (V)	J_{sc} (mA cm^{-2})	FF (%)	PCE (%)
D18:L8-BO	0.01 wt% **[10]CPP**	0.910	26.24	78.07	18.64

2.6 PL emission of the active layer

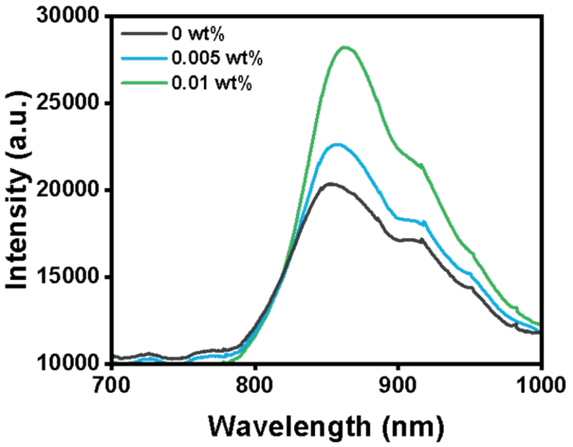

Figure S7. PL emission of the active layer with different concentrations of **[4]OMe-Pyr-[8]CPP**.

2.7 EQE curves of [n]OMe-Pyr-[8]CPP

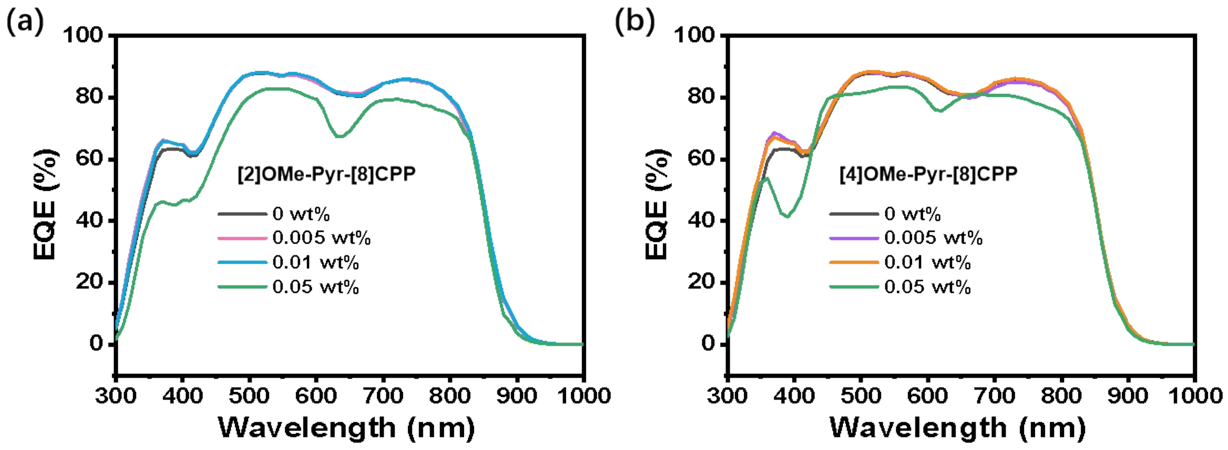

Figure S8. EQE curves of (a) **[2]OMe-Pyr-[8]CPP** and (b) **[4]OMe-Pyr-[8]CPP**.

2.8 The stability performance of OSCs

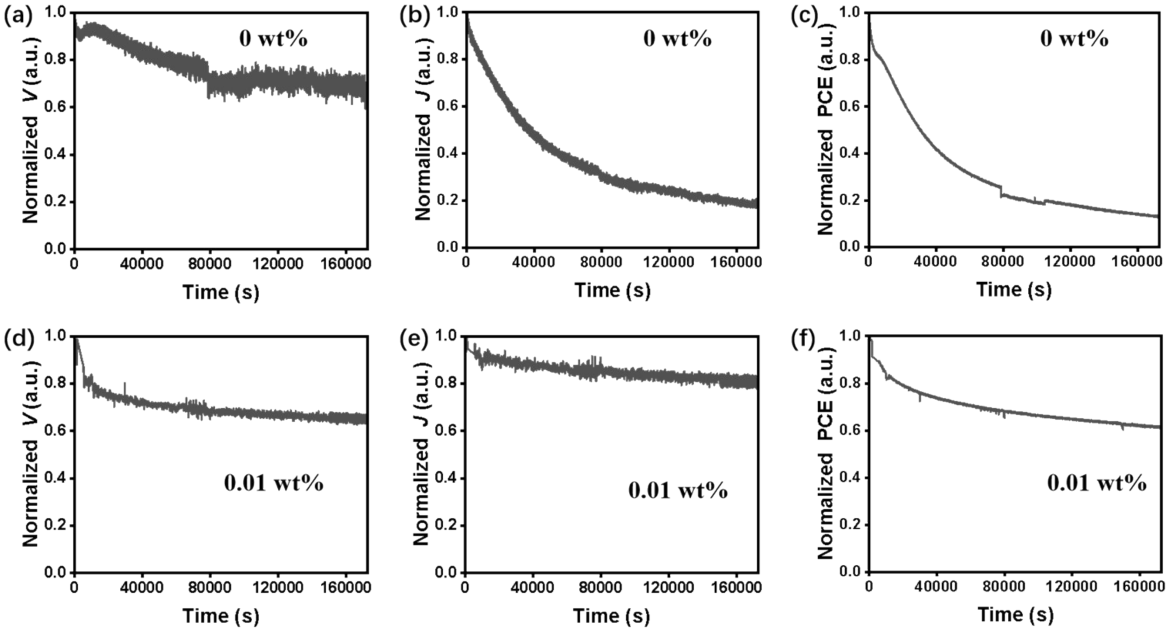

Figure S9. Light stability characterizations of photovoltaic parameters V_{oc}, J_{sc}, and PCE for devices without (a)-(c) and with **[4]OMe-Pyr-[8]CPP** dopant (d)-(f), respectively.

2.9 GIWAXS images

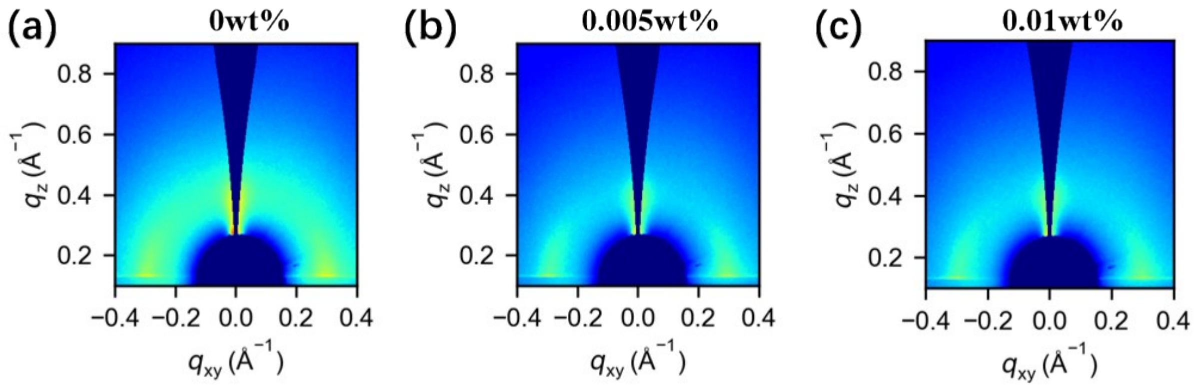

Figure S10. GIWAXS patterns of D18:L8-BO films with (a) 0 wt%, (b) 0.005 wt%, and (c) 0.01 wt% **[4]OMe-Pyr-[8]CPP** dopant.

2.10 UV-vis spectra of L8-BO

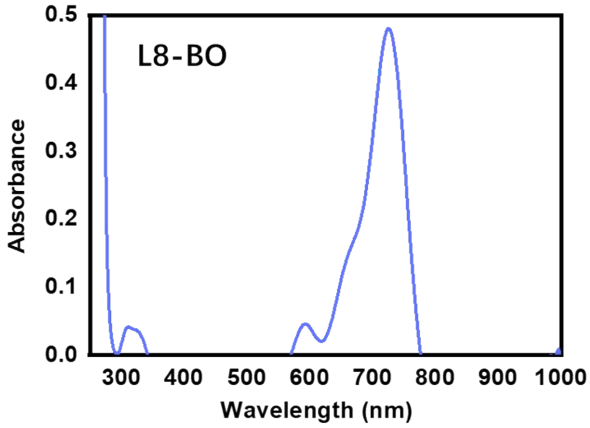

Figure S11. Absorption spectra of L8-BO in dichloromethane (c = 2.5 × 10^{-6} M).

2.11 The universality of the dopant

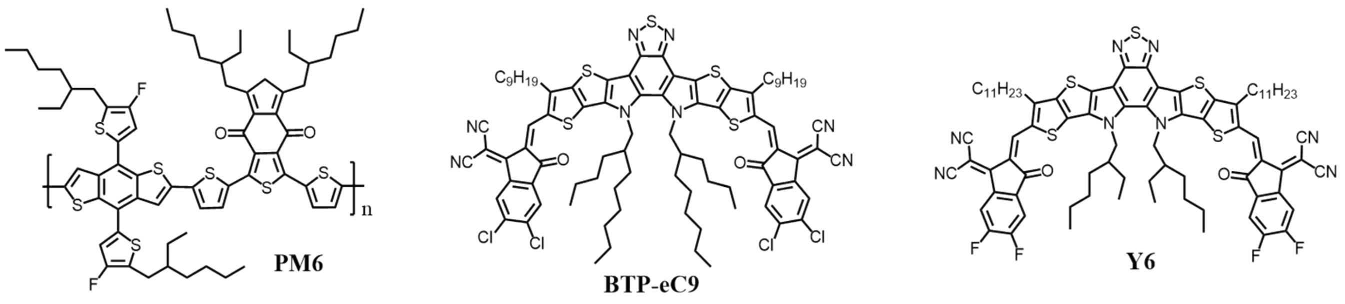

Figure S12. Chemical structures of PM6, BTP-eC9, and Y6.

PM6:BTP-eC9 system

The structure of OSCs：ITO/PEDOT:PSS/PM6:BTP-eC9/PDINN/Ag

Table S6. Device parameters of PM6:BTP-eC9-based OSCs containing different concentrations of the **[4]OMe-Pyr-[8]CPP** dopant.

Device	V_{oc} (V)	$J_{sc}/J_{cal.}$ (mA cm^{-2})	FF (%)	PCE$_{max}$/PCE$_{ave}$ (%)
PM6: BTP-eC9	0.855	27.30 (26.45)	74.39	17.80/17.15
0.005 wt% **[4]OMe-Pyr-[8]CPP**	0.858	28.06 (26.65)	71.67	17.49/17.22
0.01 wt% **[4]OMe-Pyr-[8]CPP**	0.856	29.50 (26.68)	72.02	18.44/17.88
0.02 wt% **[4]OMe-Pyr-[8]CPP**	0.853	28.90 (26.64)	71.70	17.92/17.63

PM6:Y6 system

The structure of OSCs：ITO/PEDOT:PSS/PM6:Y6/PDINN/Ag

Table S7. Device parameters of PM6:Y6-based OSCs containing different concentrations of the **[4]OMe-Pyr-[8]CPP** dopant.

Device	V_{oc} (V)	$J_{sc}/J_{cal.}$ (mA cm^{-2})	FF (%)	PCE$_{max}$/PCE$_{ave}$ (%)
PM6: Y6	0.842	28.67 (25.56)	69.33	16.75/16.73
0.005 wt% **[4]OMe-Pyr-[8]CPP**	0.841	29.56 (25.80)	68.47	17.12/17.05
0.01 wt% **[4]OMe-Pyr-[8]CPP**	0.845	29.68 (26.16)	69.44	17.51/17.41
0.02 wt% **[4]OMe-Pyr-[8]CPP**	0.850	29.26 (26.01)	67.35	17.12/17.07

2.12 Computational details and results

All theoretical calculations were performed by the density functional theory (DFT)[S8] and time-dependent DFT (TD DFT)[S9] with B3LYP functional in dichloromethane.[S10] The geometries of ground state of **[2]OMe-Pyr-[8]CPP**、**[4]OMe-Pyr-[8]CPP** were optimized at the B3LYP/6-31G (d, p) level. The absorption maximum (λ_{max}) and oscillator strength (*f*) were predicted at the TD B3LYP/6-31G (d, p) level. The conductor-like polarizable continuum model (CPCM) with dielectric constant of 8.93 were employed to consider the effect of the polar solvent dichloromethane. All of these calculations were performed by Gaussian09 program package.[S11]

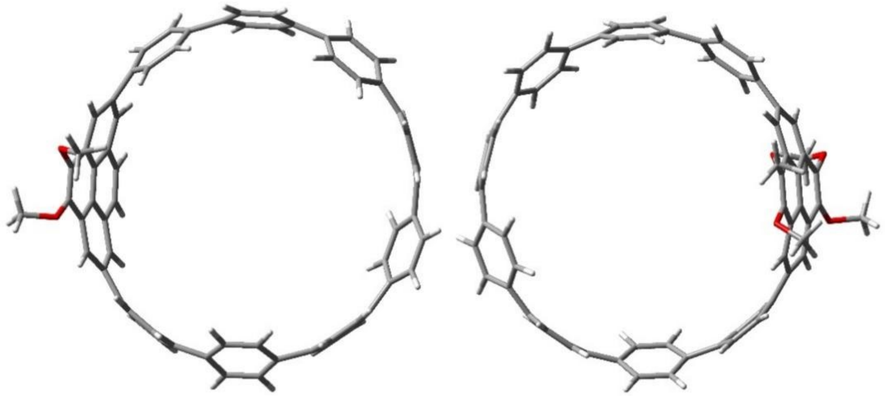

Figure S13. Optimized geometries of **[2]OMe-Pyr-[8]CPP** (left) and **[4]OMe-Pyr-[8]CPP** (right) in dichloromethane.

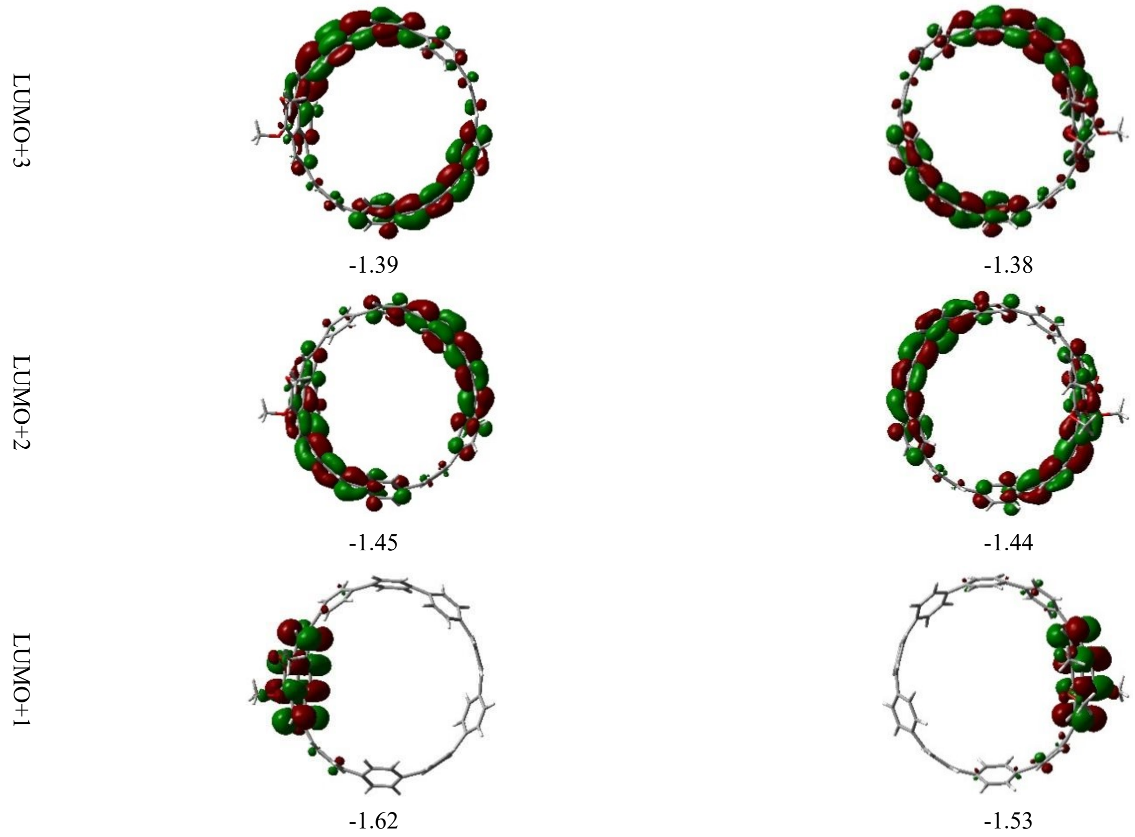

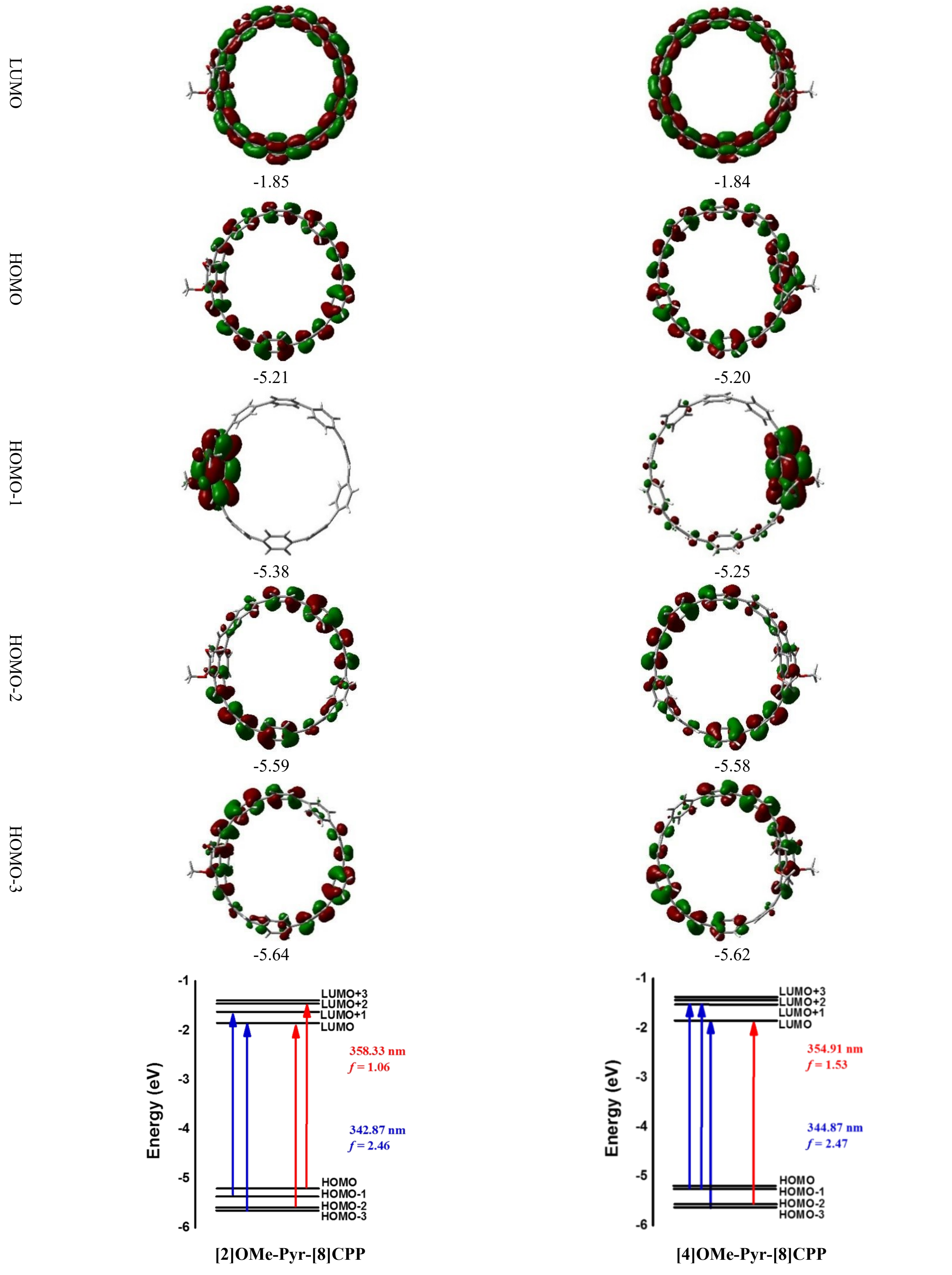

Figure S14. The TD B3LYP/6-31G (d, p) predicted frontier molecular orbital and energies (eV) of **[2]OMe-Pyr-[8]CPP** and **[4]OMe-Pyr-[8]CPP** in dichloromethane. Energy diagrams (bottom) of them are shown. Values of *f* represent the oscillator strengths.

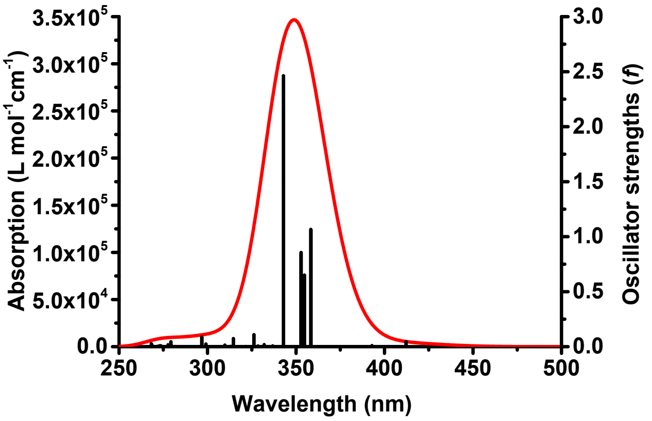

Figure S15. Simulated UV-vis spectra of **[2]OMe-Pyr-[8]CPP** in dichloromethane.

Table S8. The TD B3LYP/6-31G (d, p) calculated absorption maxima (λ_{max}, nm), oscillator strengths (*f*), and major transition in dichloromethane of **[2]OMe-Pyr-[8]CPP**.

state	Energy	λ_{max}	*f*	Description
S_3	3.4601	358.33	1.0654	HOMO-3→LUMO(0.0496)
				HOMO-2→LUMO (0.354)
				HOMO-1→LUMO+1(0.245)
				HOMO→LUMO+2(0.352)
S_4	3.4955	354.70	0.6501	HOMO-3→LUMO(0.103)
				HOMO-2→LUMO(0.046)
				HOMO-1→LUMO (0.245)
				HOMO-1→LUMO+1(0.095)
				HOMO→LUMO+1(0.418)
				HOMO→LUMO+3(0.093)
S_5	3.5135	352.88	0.8552	HOMO-3→LUMO(0.159)
				HOMO-2→LUMO(0.169)
				HOMO-1→LUMO(0.155)
				HOMO-1→LUMO+1(0.125)
				HOMO→LUMO+1(0.178)
				HOMO→LUMO+2(0.026)
				HOMO→LUMO+3(0.187)

S_6	3.6161	342.87	2.4619	HOMO-3→LUMO(0.261)
				HOMO-1→LUMO+1(0.493)
				HOMO→LUMO+2(0.118)
				HOMO→LUMO+3(0.127)
S_{10}	3.8010	326.19	0.1081	HOMO-3→LUMO+1(0.097)
				HOMO-2→LUMO+1 (0.228)
				HOMO-1→LUMO+2(0.256)
				HOMO-1→LUMO+3(0.417)

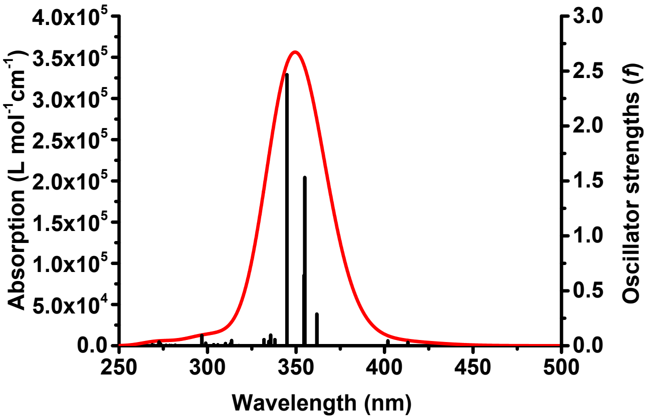

Figure S16. Simulated UV-vis spectra of **[4]OMe-Pyr-[8]CPP** in dichloromethane.

Table S9. The TD B3LYP/6-31G (d, p) calculated absorption maxima (λ_{max}, nm), oscillator strengths (*f*), and major transition in dichloromethane of **[4]OMe-Pyr-[8]CPP**.

state	Energy	λ_{max}	*f*	Description
S_3	3.4274	361.74	0.2878	HOMO-3→LUMO(0.106)
				HOMO-2→LUMO(0.202)
				HOMO-1→LUMO+1(0.413)
				HOMO-1→LUMO+2(0.050)
				HOMO→LUMO+1(0.194)
				HOMO→LUMO+3(0.035)
S_4	3.4934	354.91	1.5308	HOMO-3→LUMO(0.084)
				HOMO-2→LUMO (0.310)

				HOMO-1→LUMO(0.06)
				HOMO-1→LUMO+3(0.031)
				HOMO→LUMO+1(0.089)
				HOMO→LUMO+2(0.194)
				HOMO→LUMO+3(0.113)
S_5	3.4975	354.49	0.6386	HOMO-3→LUMO(0.101)
				HOMO-3→LUMO+1(0.291)
				HOMO-2→LUMO(0.040)
				HOMO-1→LUMO(0.194)
				HOMO-1→LUMO+1(0.215)
				HOMO→LUMO(0.237)
				HOMO→LUMO+1(0.299)
				HOMO→LUMO+3(0.099)
S_6	3.5950	344.87	2.4659	HOMO-3→LUMO(0.305)
				HOMO-2→LUMO+1(0.413)
				HOMO-1→LUMO+1(0.326)
				HOMO-1→LUMO+2(0.095)
				HOMO→LUMO+1 (0.045)
				HOMO→LUMO+2 (0.050)
				HOMO→LUMO+3 (0.138)

Cartesian coordinates

Optimized S_0 geometry of compound **[2]OMe-Pyr-[8]CPP**.

Center Number	Atomic Number	Atomic Type	Coordinates (Angstroms) X	Y	Z
1	8	0	-7.059807	-1.106508	2.303102
2	8	0	-6.881913	1.711886	2.338286

3	6	0	4.128395	-6.150087	1.396897
4	1	0	3.772252	-6.367185	2.399744
5	6	0	3.379890	-6.568115	0.279686
6	6	0	-5.484307	3.042729	0.265305
7	1	0	-5.690785	3.563738	1.193674
8	6	0	-5.585414	-0.451966	-1.030289
9	6	0	-4.117791	-4.918039	0.768601
10	1	0	-4.604496	-4.432967	1.608144
11	6	0	-4.766007	3.683123	-0.760946
12	6	0	-3.086535	-5.810630	1.039681
13	1	0	-2.839218	-6.014492	2.077122
14	6	0	5.103508	-5.574289	-1.132572
15	1	0	5.469903	-5.370952	-2.134600
16	6	0	-5.087880	-1.183603	-2.147558
17	6	0	7.563288	-1.079815	-0.169636
18	6	0	-6.023490	-1.163449	0.124438
19	6	0	-6.448073	0.969455	1.263483
20	6	0	-2.293941	-6.347274	0.007183
21	6	0	3.985655	6.228077	0.350405
22	6	0	-6.552449	-0.400192	1.234095
23	6	0	-5.514561	0.967482	-1.018975
24	6	0	-4.870709	-2.560531	-2.016972
25	1	0	-4.367018	-3.066974	-2.833328
26	6	0	-4.617463	0.908330	-3.311926
27	6	0	-4.433968	-4.536369	-0.550044
28	6	0	-0.203996	-6.308211	1.360652
29	1	0	-0.728997	-5.738223	2.119586
30	6	0	-0.909107	-6.825576	0.257581
31	6	0	-2.188706	5.834027	0.913323
32	1	0	-1.661628	5.801002	1.861878
33	6	0	1.232900	-7.612399	-0.617063
34	1	0	1.772481	-8.139746	-1.399071
35	6	0	5.719448	-4.964686	-0.022894
36	6	0	-0.158760	-7.559324	-0.682751
37	1	0	-0.659718	-8.047468	-1.514208

38	6	0	-4.951795	1.661505	-2.130269
39	6	0	-5.129089	-3.242810	-0.814226
40	6	0	-5.853646	1.693922	0.160907
41	6	0	-5.808978	-2.546806	0.204230
42	1	0	-6.093053	-3.048068	1.121852
43	6	0	6.585585	-3.766372	-0.171401
44	6	0	1.181434	-6.362643	1.427149
45	1	0	1.676899	-5.833612	2.234469
46	6	0	2.625832	6.797126	0.165325
47	6	0	-4.679582	-0.451813	-3.319336
48	6	0	-3.934849	4.891701	-0.502799
49	6	0	1.814123	7.182812	1.249566
50	1	0	2.263002	7.358529	2.223547
51	6	0	1.944654	-6.936314	0.393523
52	6	0	4.233552	5.428688	1.480858
53	1	0	3.515315	5.413312	2.294947
54	6	0	-3.498839	5.751119	-1.529562
55	1	0	-4.002961	5.742159	-2.492200
56	6	0	0.620642	6.766408	-1.220091
57	1	0	0.175505	6.540123	-2.184291
58	6	0	5.263369	-5.358654	1.249495
59	1	0	5.732315	-4.952493	2.140873
60	6	0	6.976718	3.144917	0.289255
61	6	0	-8.482605	-0.995800	2.472998
62	1	0	-9.006676	-1.353058	1.578839
63	1	0	-8.772836	0.038133	2.682461
64	1	0	-8.736297	-1.631513	3.323641
65	6	0	0.430034	7.262423	1.120350
66	1	0	-0.166675	7.499753	1.996908
67	6	0	-1.631575	6.538033	-0.169725
68	6	0	3.956048	-6.347627	-0.985959
69	1	0	3.442361	-6.688308	-1.880053
70	6	0	-6.153729	1.516221	3.563421
71	1	0	-5.092466	1.750223	3.419328
72	1	0	-6.258662	0.490453	3.925849

73	1	0	-6.586515	2.213233	4.283736
74	6	0	-0.206772	6.955227	-0.097814
75	6	0	-2.373263	6.555766	-1.366941
76	1	0	-2.029655	7.153842	-2.206616
77	6	0	6.835631	-1.597002	-1.257571
78	1	0	6.527264	-0.938637	-2.062824
79	6	0	7.679465	0.394176	-0.022237
80	6	0	-4.597220	3.008343	-1.981950
81	1	0	-4.050979	3.486792	-2.789404
82	6	0	7.927535	-1.992507	0.839802
83	1	0	8.550108	-1.663969	1.667588
84	6	0	4.959455	6.213185	-0.666102
85	1	0	4.852234	6.869480	-1.525732
86	6	0	-2.755771	-6.140735	-1.306738
87	1	0	-2.216684	-6.572503	-2.144673
88	6	0	2.004118	6.690713	-1.091978
89	1	0	2.592969	6.409409	-1.960043
90	6	0	7.589289	1.256375	-1.131583
91	1	0	7.714155	0.862562	-2.135945
92	6	0	5.291999	4.528547	1.510834
93	1	0	5.357402	3.843326	2.349505
94	6	0	7.235082	2.592812	-0.980327
95	1	0	7.056320	3.182704	-1.873954
96	6	0	7.284729	2.337549	1.401243
97	1	0	7.183662	2.735811	2.406451
98	6	0	6.361019	-2.901624	-1.258638
99	1	0	5.701326	-3.207705	-2.063859
100	6	0	-3.312078	5.030515	0.750664
101	1	0	-3.622150	4.399652	1.577900
102	6	0	7.615041	0.993894	1.249824
103	1	0	7.719634	0.382373	2.141147
104	6	0	7.450523	-3.302607	0.839086
105	1	0	7.715091	-3.955570	1.666397
106	6	0	6.026344	5.317672	-0.630784
107	1	0	6.724685	5.307250	-1.462778

108	6	0	6.165340	4.384563	0.416244
109	6	0	-3.808005	-5.270368	-1.576658
110	1	0	-4.072253	-5.093720	-2.614547
111	1	0	-4.253398	1.447514	-4.182847
112	1	0	-4.366165	-1.012418	-4.196470

Optimized S_0 geometry of compound **[4]OMe-Pyr-[8]CPP**.

Center Number	Atomic Number	Atomic Type	Coordinates (Angstroms) X	Y	Z
1	8	0	6.607082	-1.044771	-3.085194
2	8	0	6.391223	1.771332	-3.103963
3	8	0	4.236125	1.747140	3.773722
4	8	0	4.380662	-1.152154	3.856085
5	6	0	-4.513024	-6.162210	-1.433213
6	1	0	-4.220735	-6.373008	-2.457822
7	6	0	-3.685171	-6.567855	-0.368789
8	6	0	5.119504	3.078401	-0.938729
9	1	0	5.245956	3.599931	-1.881019
10	6	0	5.397217	-0.406198	0.353401
11	6	0	3.759071	-4.838094	-1.361114
12	1	0	4.184407	-4.346883	-2.229938
13	6	0	4.460358	3.700971	0.134523
14	6	0	2.710115	-5.728388	-1.562622
15	1	0	2.387837	-5.925524	-2.580654
16	6	0	-5.327097	-5.603217	1.156362
17	1	0	-5.629035	-5.405940	2.180843
18	6	0	4.993095	-1.136254	1.505611
19	6	0	-7.902204	-1.141976	0.360360
20	6	0	5.742655	-1.113564	-0.833500
21	6	0	6.051463	1.023482	-1.999187
22	6	0	1.995009	-6.273100	-0.479032
23	6	0	-4.395811	6.170589	-0.399427
24	6	0	6.176904	-0.344171	-1.979240
25	6	0	5.294588	1.009487	0.346904
26	6	0	4.728119	-2.505409	1.375137
27	1	0	4.245623	-3.001245	2.207778
28	6	0	4.601533	0.953977	2.729184
29	6	0	4.171601	-4.466703	-0.066353
30	6	0	-0.185048	-6.245403	-1.682884

31	1	0	0.279841	-5.663206	-2.471241
32	6	0	0.599929	-6.761232	-0.634570
33	6	0	1.738621	5.810456	-1.358955
34	1	0	1.151137	5.771861	-2.271111
35	6	0	-1.467301	-7.582553	0.377741
36	1	0	-1.945580	-8.122307	1.190572
37	6	0	-6.024985	-5.003129	0.090854
38	6	0	-0.075225	-7.510245	0.349656
39	1	0	0.487594	-7.996739	1.141587
40	6	0	4.797690	1.693083	1.491539
41	6	0	4.899716	-3.183461	0.154045
42	6	0	5.526617	1.739067	-0.856276
43	6	0	5.522863	-2.496814	-0.904249
44	1	0	5.741394	-2.997005	-1.840068
45	6	0	-6.895926	-3.817045	0.297954
46	6	0	-1.570902	-6.319266	-1.656270
47	1	0	-2.127065	-5.792034	-2.424290
48	6	0	-3.027989	6.742112	-0.302230
49	6	0	4.699678	-0.418602	2.729827
50	6	0	3.587266	4.891198	-0.062725
51	6	0	-2.289082	7.130284	-1.436400
52	1	0	-2.800479	7.304008	-2.379433
53	6	0	-2.254778	-6.911690	-0.578534
54	6	0	-4.713157	5.367815	-1.509917
55	1	0	-4.047503	5.351114	-2.367550
56	6	0	3.205759	5.740958	0.993374
57	1	0	3.771007	5.736228	1.921374
58	6	0	-0.938051	6.719281	0.951639
59	1	0	-0.431034	6.495165	1.885297
60	6	0	-5.648481	-5.389466	-1.209569
61	1	0	-6.182043	-4.990429	-2.067195
62	6	0	-7.371739	3.083554	-0.141971
63	6	0	8.008958	-0.912400	-3.371515
64	1	0	8.610428	-1.253533	-2.520802
65	1	0	8.263965	0.124173	-3.611589

66	1	0	8.203124	-1.551238	-4.235421
67	6	0	-0.899966	7.216099	-1.396135
68	1	0	-0.361735	7.456328	-2.309010
69	6	0	1.244677	6.506536	-0.240675
70	6	0	-4.179540	-6.357728	0.932688
71	1	0	-3.601850	-6.690450	1.790001
72	6	0	5.567744	1.570659	-4.266317
73	1	0	4.518678	1.788685	-4.034598
74	1	0	5.656578	0.548029	-4.641955
75	1	0	5.930221	2.276829	-5.015956
76	6	0	-0.185001	6.912295	-0.221213
77	6	0	2.061184	6.530339	0.906630
78	1	0	1.764381	7.121517	1.768782
79	6	0	-7.097477	-1.649364	1.397468
80	1	0	-6.742816	-0.986160	2.179411
81	6	0	-8.040530	0.330619	0.218481
82	6	0	4.394610	3.027061	1.366796
83	1	0	3.902471	3.493776	2.210625
84	6	0	-8.324291	-2.060536	-0.620824
85	1	0	-9.005558	-1.740662	-1.404598
86	6	0	-5.303603	6.157450	0.676369
87	1	0	-5.143426	6.816274	1.525770
88	6	0	2.548002	-6.072000	0.799929
89	1	0	2.069565	-6.508227	1.671720
90	6	0	-2.326613	6.637706	0.912528
91	1	0	-2.857797	6.354098	1.816388
92	6	0	-7.884885	1.195643	1.318305
93	1	0	-7.943089	0.802890	2.329150
94	6	0	-5.770267	4.466500	-1.470963
95	1	0	-5.887022	3.778735	-2.301954
96	6	0	-7.547131	2.533387	1.142557
97	1	0	-7.314028	3.125738	2.021950
98	6	0	-7.747070	2.272848	-1.230534
99	1	0	-7.712036	2.669600	-2.240764
100	6	0	-6.608899	-2.948420	1.367319

101	1	0	-5.892581	-3.245542	2.126169
102	6	0	2.881077	5.022383	-1.272025
103	1	0	3.145216	4.397351	-2.119377
104	6	0	-8.061063	0.928114	-1.056174
105	1	0	-8.219532	0.314431	-1.938037
106	6	0	-7.833289	-3.365145	-0.651432
107	1	0	-8.146015	-4.022351	-1.458366
108	6	0	-6.369534	5.260618	0.710559
109	1	0	-7.014026	5.251512	1.584982
110	6	0	-6.572780	4.324500	-0.323104
111	6	0	3.617975	-5.204002	0.998597
112	1	0	3.957058	-5.032798	2.015407
113	6	0	4.455776	1.359225	5.132997
114	1	0	3.761499	0.579813	5.450156
115	1	0	4.290717	2.266901	5.717501
116	1	0	5.486674	1.019150	5.280473
117	6	0	5.479457	-1.833512	4.480824
118	1	0	5.049536	-2.424037	5.292284
119	1	0	6.200335	-1.116162	4.890869
120	1	0	5.993502	-2.496295	3.777522

2.13 ^{1}H, ^{13}C NMR and Mass Spectra

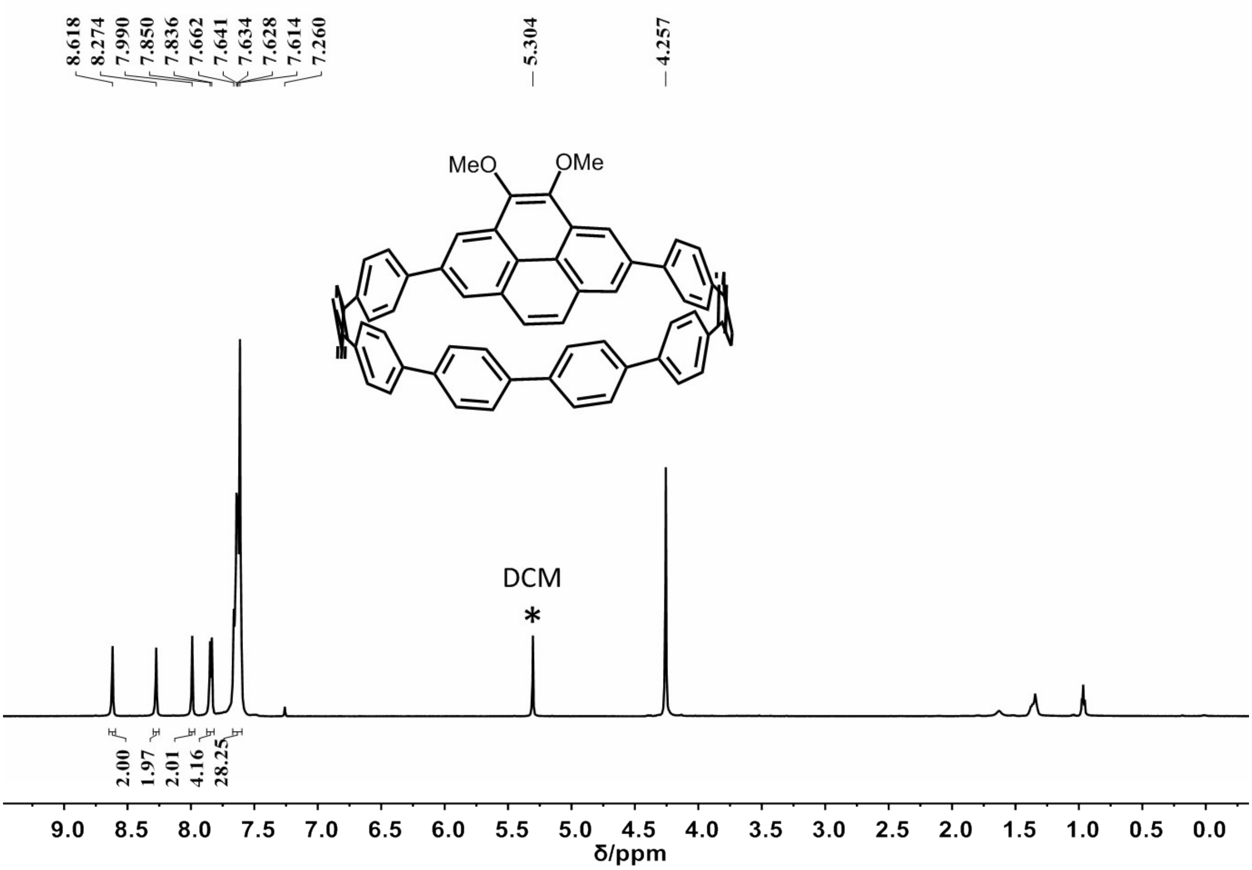

Figure S17. ^{1}H NMR spectrum of compound **[2]OMe-Pyr-[8]CPP** in $CDCl_3$ (600 MHz, 298 K).

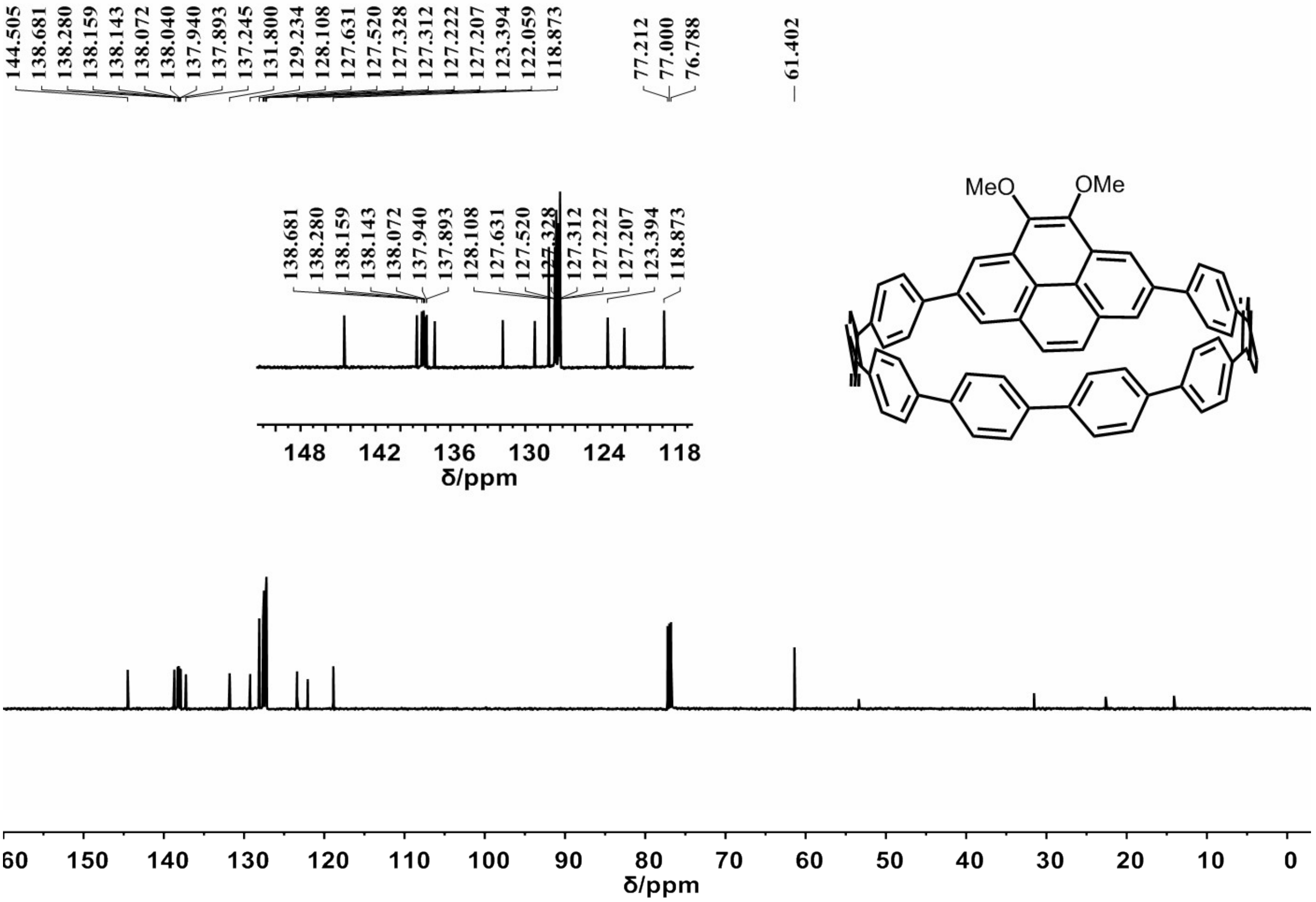

Figure S18. ^{13}C NMR spectrum of compound **[2]OMe-Pyr-[8]CPP** in $CDCl_3$ (150 MHz, 298 K).

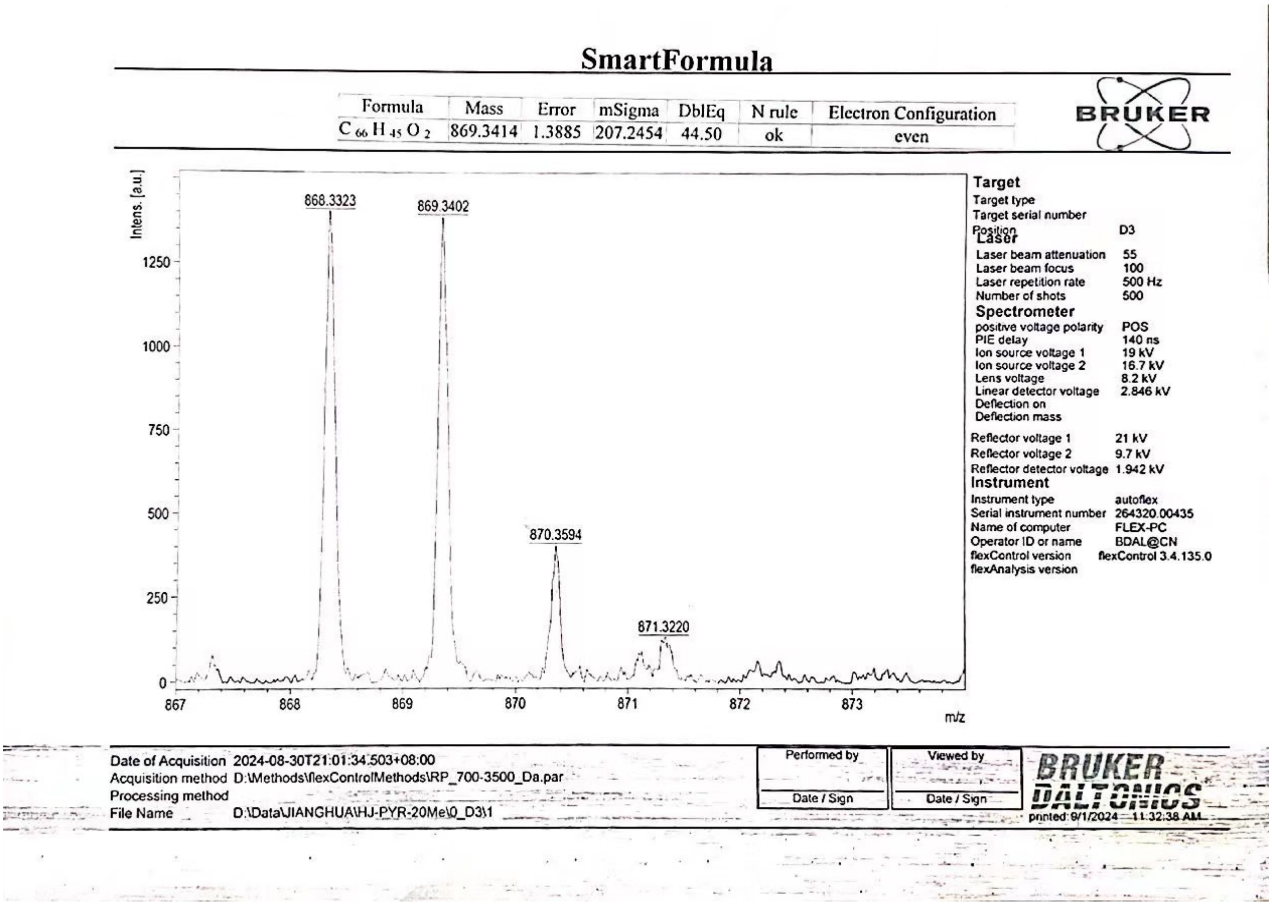

Figure S19. High-resolution mass spectrum (MALDI-TOF) of **[2]OMe-Pyr-[8]CPP**.

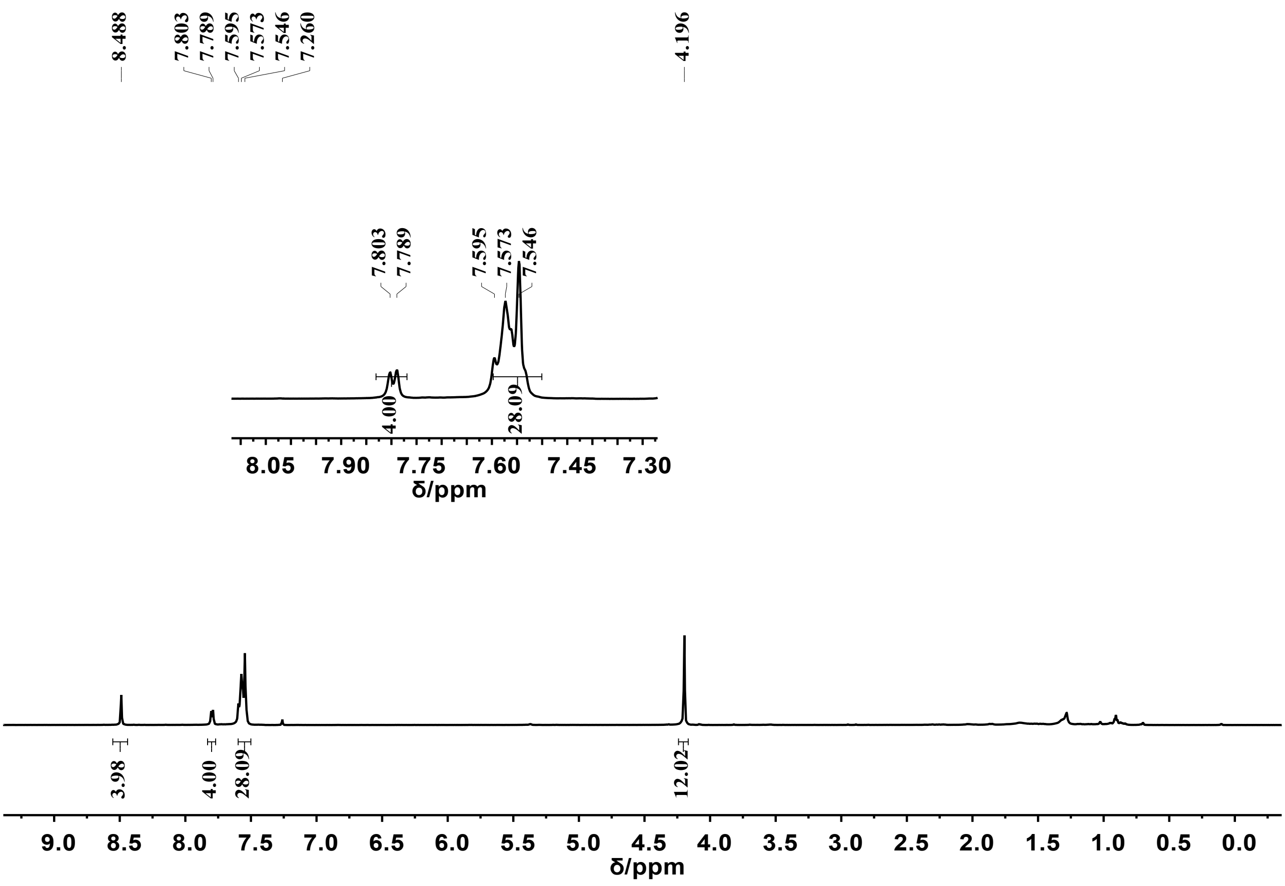

Figure S20. ^{1}H NMR spectrum of compound **[4]OMe-Pyr-[8]CPP** in $CDCl_3$ (600 MHz, 298 K).

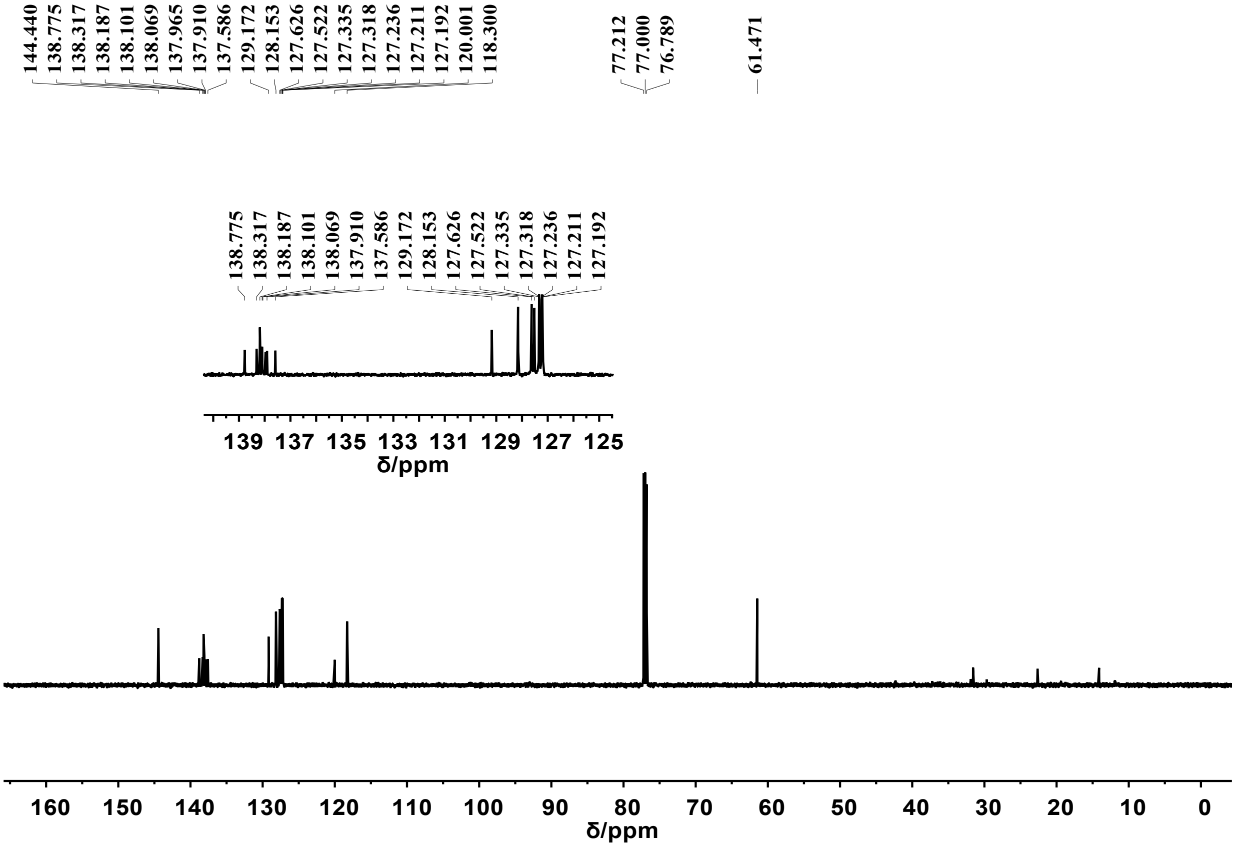

Figure S21. ^{13}C NMR spectrum of compound **[4]OMe-Pyr-[8]CPP** in $CDCl_3$ (150 MHz, 298 K).

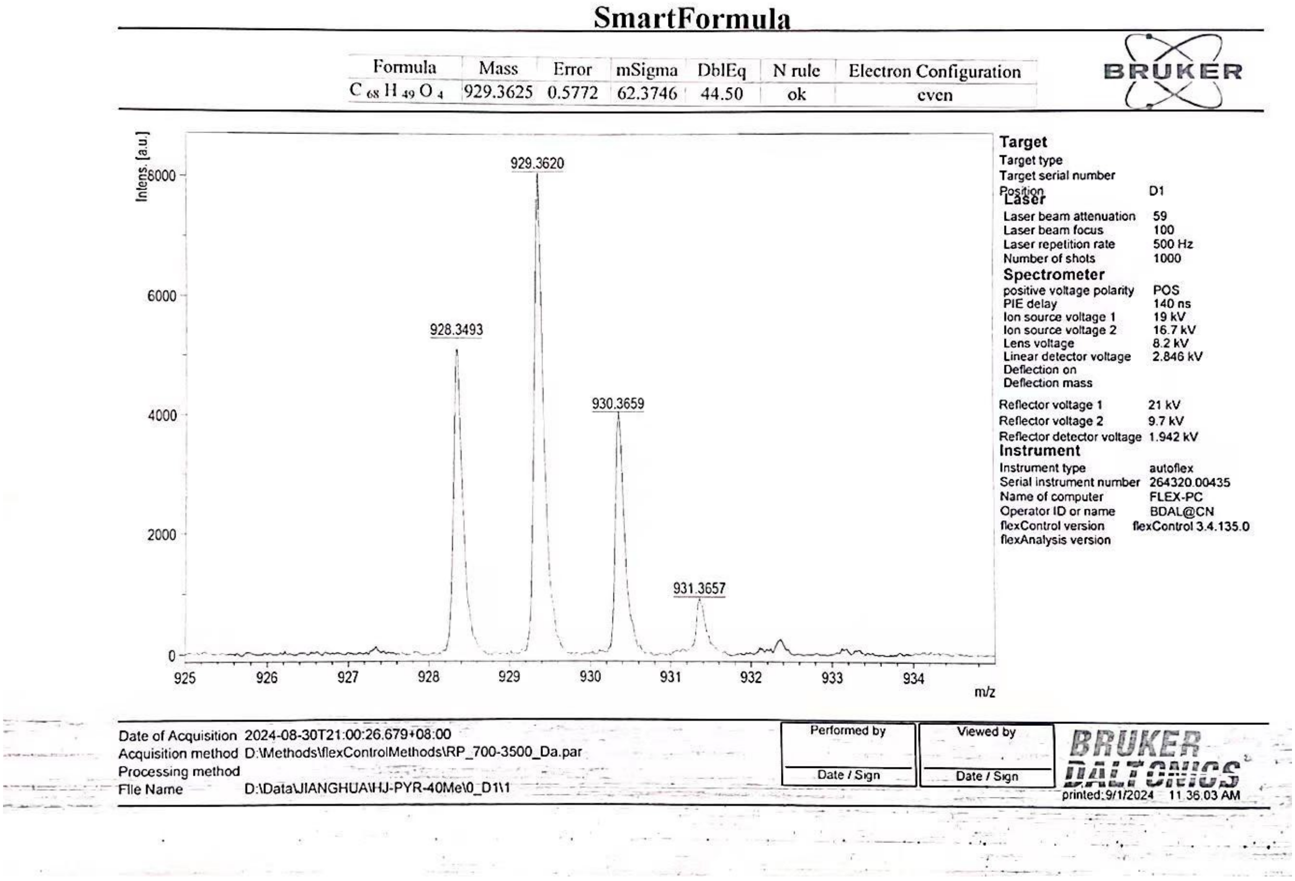

Figure S22. High-resolution mass spectrum (MALDI-TOF) of **[4]OMe-Pyr-[8]CPP**.

References

[S1] S. N. Keller, N. L. Veltri, Todd. C. Sutherland. *Org. Lett.* **2013***, 15,* 4798-4801.

[S2] S. Kawano, M. Baumgarten, D. Chercka, V. Enkelmanna, K. Müllen. *Chem. Commun.* **2013***, 49,* 5058-5060.

[S3] J. Wang, H. Shi, S. Wang, X. Zhang, P. Fang, Y. Zhou, G.-L. Zhuang, X. Shao, P. Du. *Chem. Eur. J.* **2022**, *28*, e202103828.

[S4] O. V. Dolomanov, L. J. Bourhis, R. J. Gildea, J. A. K. Howard, H. Puschmann, *J. Appl. Cryst.* **2009**, 42, 339-341.

[S5] G. M. Sheldrick, *Acta Cryst.* **2015**, *A71*, 3-8.

[S6] G. M. Sheldrick, *Acta Cryst.* **2015**, *C71*, 3-8.

[S7] A. L. Spek, *Acta Cryst.* **2015**, *C71*, 9-18.

[S8] a) J. P. Perdew, A. Ruzsinszky, J. M. Tao, V. N. Staroverov, G. E. Scuseria, G. I. Csonka, *J. Chem. Phys.* **2005,** *123***,** 062201. b) A. Dreuw, M. Head-Gordon, *Chem. Rev.* **2005**, *105*, 4009-4037.

[S9] a) R. E. Stratmann, G. E.Scuseria, M. J. Frisch, *J. Chem. Phys.* **1998**, *109*, 8218-8224. b) E. Runge, E. K. U. Gross, *Phys. Rev. Lett.* **1984**, *52*, 997-1000.

[S10] a) A. D. Becke, *Phys. Rev. A* **1988**, *38*, 3098-3100. (b) C. T. Lee, W. T. Yang, R. G. Parr, *Phys. Rev. B* **1988**, *37*, 785-789.

[S11] Gaussian 09, Revision D.01, M. J. Frisch, G. W. Trucks, H. B. Schlegel, G. E. Scuseria, M. A. Robb, J. R. Cheeseman, G. Scalmani, V. Barone, B. Mennucci, G. A. Petersson, H. Nakatsuji, M. Caricato, X. Li, H. P. Hratchian, A. F. Izmaylov, J. Bloino, G. Zheng, J. L. Sonnenberg, M. Hada, M. Ehara, K. Toyota, R. Fukuda, J. Hasegawa, M. Ishida, T. Nakajima, Y. Honda, O. Kitao, H. Nakai, T. Vreven, J. A. Jr. Montgomery, J. E. Peralta, F. Ogliaro, M. Bearpark, J. J. Heyd, E. Brothers, K. N. Kudin, V. N. Staroverov, R. Kobayashi, J. Normand, K. Raghavachari, A. Rendell, J. C. Burant, S. S. Iyengar, J. Tomasi, M. Cossi, N. Rega, M. J. Millam, M. Klene, J. E. Knox, J. B. Cross, V. Bakken, C. Adamo, J. Jaramillo, R. Gomperts, R. E. Stratmann, O. Yazyev, A. J. Austin, R. Cammi, C. Pomelli, J. W. Ochterski, R. L. Martin, K. Morokuma, V. G. Zakrzewski, G. A. Voth, P. Salvador, J. J. Dannenberg, S. Dapprich, A. D. Daniels, Ö. Farkas, J. B. Foresman, J. V. Ortiz, J. Cioslowski, D. J. Fox, Gaussian 09, Revision D.01; Gaussian, Inc.: Wallingford, CT, 2009.
